# Supplementary material for: Megafauna mobility: Assessing the foraging range of an extinct macropodid from central eastern Queensland, Australia
Source: PLoS One. 2025 Apr 23;20(4):e0319712. doi: 10.1371/journal.pone.0319712 (PMC12017834; doi:10.1371/journal.pone.0319712)
Supplement: S1 File — (DOCX) [file pone.0319712.s002.docx]

# **Megafauna mobility: assessing the foraging range of an extinct macropodid from central eastern Queensland, Australia.**

# **Study Site – Mount Etna Caves**

Fossils examined in this study were collected from three stratigraphic units (QML1311C/D, QML1311H, QML1384LU) across two large cave systems: Speaking Tube Cave and Elephant Hole Cave (S2 Fig). In the Speaking Tube Cave system, QML1311C/D and QML1311H are located at a similar level within the cave system but are separated from each other by an older deposit, QML1311F [1, 2]. Current chronology suggests age estimates of <300 ka for QML1311C/D, and >123 – 454 ka for QML1311H, based on thermal ionisation mass spectrometry U-series dating of capping/basal flowstones, bones, teeth, shell and calcite infillings [1, 2].

Elephants Hole Cave contains a number of deposits recovered throughout a vertically oriented cave system with one of the lowest deposits pertaining to QML1384LU [1]. Fossil teeth and bones buried in unlithified sediments suggests fossil deposits were emplaced by pitfall accumulation, however, small fauna fossils also indicate predator roost deposits, derived primarily from owls and ghost bats [2]**.** Stratigraphic positioning and faunal assemblages indicate that the shallower deposit of QML1312 is significantly younger (170 – 205 ka) than unit QML1384LU (>267 – 330 ka). This younger deposit contains xeric-adapted, post-rainforest species while the underlying deposit (QML1384LU) represents a similar rainforest period observed in Speaking Tube Cave [2].

# **Single-grain TT-OSL dating methods**

## *2.1 Sample collection and preparation*

Sample MTE17-1 was collected by extracting an intact block of consolidated sediment from the QML1311 C/D stockpiles. Sample MTE17-4 was collected by coring *in situ* sediment remains of QML1311H using a rotary hammer drill fitted with a 63 mm diameter coring attachment. All samples were immediately sealed with light-proof plastic upon extraction. Approximately 100 g of additional bulk sediment was collected from material directly surrounding each sample for dosimetry and water content assessments.

Quartz fractions were extracted from the unexposed portions of each luminescence dating sample under safe light conditions (630 nm LEDs, <0.15 μW/cm^2^ power density at sample position) at the University of Adelaide. The 180–250 μm fractions were prepared for burial dose estimation using standard procedures [3]. Sediment samples were initially sieved to isolate the fine sand fraction (90–300 µm). Organics and carbonates were then eliminated using concentrated (30%) hydrogen peroxide (H_2_O_2_) and hydrochloric (HCl) acid digestion. Quartz grains were isolated using heavy liquid (LST lithium heteropolytungstate) density ranges of 2.62 g/cm^3^ to 2.72 g/cm^3^. The 180–250 µm quartz fractions were then sieved and etched with hydrofluoric (HF) acid to remove the alpha-irradiated external layers (48% HF digestion for 40 min). Etched grains were subsequently washed in 30% hydrochloric acid to remove any precipitated fluorides and re-sieved using a 90 µm sieve to eliminate any disaggregated grains.

## *2.2 Equivalent dose (D_e_) estimation*

Single-grain TT-OSL measurements were made on the 180–250 μm quartz fractions using two Risø TL-DA-20 readers equipped with blue LEDs (470 nm, maximum power of 92 or 102 mW/cm^2^), infrared LEDs (peak emission 850 nm, maximum power of 302 or 349 mW/cm^2^), and 10 mW Nd:YVO_4_ single-grain laser attachments emitting at 532 nm (maximum power of ~50 W/cm^2^). Ultraviolet TT-OSL signals were detected using EMI 9235QA photomultiplier tubes fitted with 7.5 mm-thick Hoya U-340 filters. Samples were irradiated with mounted ^90^Sr/^90^Y beta sources that had been calibrated to administer known doses to multi-grain aliquots and single-grain discs (average single-grain dose rates at the time of measurement = 0.09 Gy/s). For single-grain measurements, spatial variations in the beta dose rates across the disc plane were taken into account by undertaking hole-specific calibrations using gamma-irradiated quartz.

The TT-OSL D_e_ determination procedures employed in this study are based on Arnold et al. [4], [5] and Demuro et al. [6], [7]. Single-grain quartz D_e_ measurements were undertaken using standard single-grain aluminium discs drilled with an array of 300 μm x 300 μm holes. Single-grain TT-OSL D_e_ values have been obtained using the single-aliquot regenerative dose (SAR) protocol shown in Table S3. A total of 500 single-grain D_e_ measurements were made for each sample. Single-grain TT-OSL D_e_ values were calculated by integrating the first 0.17 s of stimulation and subtracting a late-light background from the last 0.25 s. The single-grain TT-OSL dose-response curves are generally characterised by continued signal growth at high doses (10^2^-10^3^ Gy) and are typically well-represented by a single saturating exponential function (e.g. S3 Fig).

Single-grain TT-OSL D_e_ estimates were only included in the final age calculations if they satisfied a series of standard quality assurance criteria (Table H in S1 Table). Single-grain TT-OSL D_e_ estimates were rejected from further consideration if they exhibited one or more of the following properties: (i) weak TT-OSL signals (i.e., the net intensity of the natural test-dose signal, T_n_, was less than three times the standard deviation of the late-light background signal); (ii) poor recycling ratios (i.e., the ratios of sensitivity-corrected luminescence response (L_x_/T_x_) for two identical regenerative doses were not consistent with unity at 2σ); (iii) high levels of signal recuperation (i.e., the sensitivity-corrected luminescence response of the 0 Gy regenerative-dose point amounted to more than 5% of the sensitivity-corrected natural signal response (L_n_/T_n_) at 2σ); (iv) poorly defined or non-monotonic dose-response curves (i.e., those displaying no discernible dose-response curve, or a zero (flat) or negative response with increasing dose) and dose-response curves displaying very scattered L_x_/T_x_ values (i.e., those that could not be successfully fitted with the Monte Carlo procedure and, hence, did not yield finite D_e_ values and uncertainty ranges); (v) saturated or non-intersecting natural TT-OSL signals (i.e., L_n_/T_n_ values equal to, or greater than, the *I_max_* saturation limit of the dose-response curve at 2σ); (vi) contamination by feldspar grains or inclusions (i.e., the ratio of the L_x_/T_x_ values obtained from two identical regenerative doses measured with and without prior IR stimulation (OSL IR depletion ratio; Duller [8]) was less than unity at 2σ). In the case of criterion (vi), feldspar contamination was checked by measuring the OSL IR depletion ratio separately and in the standard manner for single-grain OSL measurements, i.e., by measuring two conventional single-grain OSL SAR cycles (as opposed to two single-grain TT-OSL SAR cycles) with and without IR stimulation. The TT-OSL grain classification statistics obtained for each sample after applying these quality assurance criteria are summarised in Table H, S1 Table.

Individual D_e_ estimates are presented with their 1σ error ranges, which are derived from three sources of uncertainty: (i) a random uncertainty term arising from photon counting statistics for each OSL or TT-OSL measurement, calculated using Eq. 3 of Galbraith [9]; (ii) an empirically determined instrument reproducibility uncertainty of 1.5 or 1.9% for each single-grain measurement (calculated for the specific Risø reader used for each sample using the approach outlined in Jacobs et al. [10]); and (iii) a dose-response curve fitting uncertainty determined using 1000 iterations of the Monte Carlo method described by Duller [11] and implemented in Analyst v4.

## *2.3 SAR D_e_ validation tests*

The suitability of the single-grain TT-OSL SAR protocol (Table C in S1 Table) was assessed by performing a dose recovery test on sample MTE17-4. For this purpose, a laboratory dose of ~374 ± 7 Gy was added on top of the naturally accumulated dose for a subset of 500 grains (unbleached and dosed grains). This approach was adopted owing to the long durations of light exposure needed to bleach natural TT-OSL signals down to low residual levels [e.g., 5, 7]. The dose recovery ratio was calculated by first adding the weighted mean natural TT-OSL D_e_ of sample MTE17-4 (i.e., 375 ± 23 Gy, as shown in Table 1 and determined from 500 grains) to the weighted mean administered dose of the accepted grain population from the dose recovery test (374 ± 7 Gy). This was used to constrain the combined administered (natural + laboratory) dose being assessed in the dose recovery test. The measured (recovered) weighted mean D_e_ of the unbleached and dosed grains in the dose recovery test (750 ± 35 Gy; S3 Fig) was then divided by the combined administered (natural + laboratory) dose (750 ± 24 Gy) to derive the measured-to-given dose ratio. A weighted mean TT-OSL measured-to-given dose ratio of 1.00 ± 0.06 was obtained using this approach, which supports the suitability of the chosen measurement conditions for dating these samples.

## *2.4 Dose rate determination*

Table 1 and Table H in S1 Table summarises the dose rate estimates for the TT-OSL dating samples. For sample MTE17-4, the external gamma and beta dose rates have been calculated using a combination of *in situ* field gamma-ray spectrometry and low-level beta counting. Field gamma spectrometry measurements were performed at the luminescence dating sample position and elemental concentrations of K, U, and Th were determined from the field gamma-ray spectra using the ‘energy windows’ method described in Arnold et al. [12] and Duval and Arnold [13]. These elemental concentrations were then used to calculate the gamma dose rates, enabling us to capture any spatial heterogeneity in the surrounding (~30 cm diameter) gamma radiation field of each sample. External beta dose rates were determined from measurements made using a Risø GM-25-5 beta counter [14] on dried and homogenised, bulk sediments collected directly from the luminescence dating sampling position. This approach was used to ensure that beta dose rates were derived from sample sizes that closely approximate the very short (~2–3 mm) beta particle radiation fields affecting this sample. Background-subtracted count rates were measured for three aliquots of each sample and compared with net count rates obtained simultaneously for a loess sediment standard with known U, Th, and K concentrations [15].

Unfortunately, it was not possible to undertake *in situ* gamma spectrometry measurements at the time of sample collection for sample MTE17-1. The beta and gamma dose rates of this sample was therefore both determined in the laboratory using high-resolution gamma spectrometry (HRGS) measurements made on dried and homogenised, bulk sediments collected directly from the TT-OSL sampling positions. HRGS measurements were conducted using a p-type, high-purity, germanium well detector at the University of Adelaide, following the procedures outlined in Leslie [16]. ~8 g of sediment sample was sealed in a plastic container for at least 30 days (the equivalent of ~8 half-lives of ^222^Rn; t_1/2_ = 3.825 days) to enable the post-radon daughters of ^214^Pb and ^214^Bi to build up and reach equilibrium with parental ^226^Ra activities. Following re-establishment of equilibrium in the post-radon nuclides, we counted the sealed sample in the detector for 4 days in near-4π geometry to maximise detector efficiency. The HRGS radionuclide activities obtained for sample MTE17-1 are summarised in Table I of S1 Table. The ^238^U and ^232^Th decay chains of this sample are in present-day secular equilibrium; i.e., the daughter-parent isotopic ratios for ^238^U, ^226^Ra, ^210^Pb, ^228^Ra and ^228^Th are consistent with unity at 1σ. Representative dose rates have been calculated from the HRGS radionuclide concentrations after considering the proportional beta and gamma dose rate contributions from different parent or daughter isotopes measured in the ^238^U and ^232^Th decay series. As part of this analysis, the present-day state of equilibrium observed in the ^238^U and ^232^Th decay chains are assumed to have prevailed throughout the burial period.

Cosmic-ray dose rates were calculated using the approach described in Prescott and Hutton [17], taking into consideration site altitude, geomagnetic latitude, and density, thickness and geometry of sediment overburden. The effects of bedrock shielding have additionally been incorporated into the cosmic dose rate calculation using the present-day rock shelter geometry and the integrated cos^2^ ϕ-zenith angular dependence procedures outlined in Allkofer [18] and Smith et al. [19].

A small, assumed internal (alpha plus beta) dose rate of 0.03 ± 0.01 Gy / ka has been included in the final dose rate calculations for these samples, based on published ^238^U and ^232^Th measurements for etched quartz grains from a range of locations [e.g., 20, 21-24] and an alpha efficiency factor (a-value) of 0.04 ± 0.01 [25, 26].

Radionuclide concentrations and specific activities have been converted to dose rates using the conversion factors given in Readhead [27] and Guérin et al. [28] (see footnotes of Table 1 for details), making allowance for beta-dose attenuation [29, 30] and long-term sediment water contents [31, 32]. The present-day sediment water content of sample MTE17-4 (15.7% dry sediment weight) is considered to be broadly representative of moisture conditions prevailing through the sample burial period because in situ sediment deposits were targeted for sampling. As such, the present-day sediment water contents of MTE17-4 has been used in the final dose rate calculation.

In contrast, sample MTE17-1 yielded an uncharacteristically low present-day sediment water contents of 4.8% dry weight. This samples was collected from an isolated fragment block of QML1311 C/D recovered during stockpiling operations, which was subsequently stored outside of its natural depositional context prior to sampling for luminescence dating. The enhanced potential for desiccation of this block prior to sampling means that its empirical water content estimate is unlikely to be entirely representative of the moisture conditions prevailing throughout the sample burial period. For sample MTE17-1, a more suitable empirical long-term water content estimate has been derived from a site-specific proportional saturated water contents assessment undertaken on sample MTE17-4, which was collected from freshly exposed and better preserved deposits and is thought to more closely reflect long-term hydrological conditions for the Speaking Tube Cave. Sample MTE17-4 has a present-day water content equivalent to 35% of its saturated water content. On the basis of this proportional saturated water content assessment, we have adopted a similar long-term estimate equivalent to 35% of the present-day saturated water contents for sample MTE17-1. The final water contents of this samples is therefore 19.8% dry weight (mean ± 1σ = 13.9 ± 1.0% dry weight), overlapping with reported water contents of analogous deposits from karst systems elsewhere in Australia and globally [e.g., 4, 5, 33, 34-40]. A 1σ relative uncertainty of 20% (40% at 2σ) has been assigned to the long-term moisture estimates of MTE17-1 and MTE17-4 to accommodate any variations in hydrologic conditions during burial. These assigned 2σ uncertainty ranges ensure that the long-term sediment moisture contents span all reasonable possible hydrological scenarios between likely minimum and maximum end-member limits over past glacial and interglacial cycles.

Whilst 35% of saturated water content has been chosen for the final dose rate calculation of MTE17-1, it is worth noting that use of a lower or higher proportional saturated water content estimate would not alter the final age estimates of this sample beyond its existing uncertainty ranges. For example, use of a lower long-term water content equivalent to 20% present-day saturated values (equivalent to 11.3% dry weight) would cause the final TT-OSL ages to decrease by 23.2 ka. Similarly, adopting a higher long-term sediment moisture content of 60% present-day saturated values (equivalent to 33.9% dry weight) would cause the final TT-OSL ages to increase by 38.4 ka. In both of these extreme scenarios, the resultant TT-OSL ages are statistically indistinguishable (at 2σ) from that shown for sample MTE17-1 in Table 1, and therefore our luminescence chronologies and interpretations are considered relatively insensitive to the preferred choice of long-term water content.

## Element concentrations as a Proxy for Diagenesis

## *Element concentrations - methods*

Element concentrations were measured in-situ by LA-ICP-MS, at WIGL. Analysis was performed with an ESL™ 193 nm ArF excimer laser ablation system, equipped with a TV2 cell, coupled with a Thermo iCAP Q quadrupole ICP-MS.

Samples were pre-ablated with a spot size of 60 µm, a laser pulse rate of 5 Hz, and a fluence of 1.67 J/cm^2^, with a dwell time of 2s. Samples were then ablated with a spot size of 50 µm, a laser pulse rate of 20 Hz and a fluence of 5.3 J/cm^2^, with a dwell time of 60 s. Laser warm-up time was 10 s and washout time was 30s. Helium was used as a carrier gas at a flow rate of 800 mL/min. Analyses were carried out on enamel and dentine, across the tooth surface, examining intra-tooth variations in elemental concentration. The laser ablation system was connected to the quadrupole ICP – MS using an ESL glass smoothing device, connected to Ar sample gas from the ICP-MS. The ICP-MS was equipped with nickel sample and skimmer cones and a high sensitivity insert. The following analytes were collected on the ICP-MS: ^24^Mg, ^43^Ca, ^47^Ti, ^48^Ca, ^55^Mn, ^88^Sr, ^137^Ba, ^139^La, ^140^Ce, ^141^Pr, ^146^Nd, ^147^Sm, ^153^Eu, ^157^Gd, ^159^Tb, ^163^Dy, ^165^Ho, ^166^Er, ^169^Tm, ^172^Yb, ^175^Lu. Dwell time for each analyte was 0.1 s, except for ^24^Mg, ^43^Ca and ^48^Ca (0.01 s).

At the start of each session, the system was tuned with SRM NIST 612 glass with a spot size of 65 µm, scan speed of 5 µm/s, laser pulse rate of 5 Hz and fluence of 10.9 J/cm^2^. Before and after each sample measurement, 3 spots were ablated on two mounts of US Geological Survey (USGS) reference material, MAPS-4: (i) a pellet of MAPS-4 was used as calibration standard and (ii) sintered powder of MAPS-4 was used to assess accuracy. Data reduction was performed in Iolite 4.0™ [41], using the ‘trace elements’ data reduction scheme. Baseline was subtracted from each analyte, ^43^Ca was used as internal standard and USGS MAPS-4 reference material as external standard. All element concentrations measured on the sintered powder of USGS MAPS-4 were within error of recommended values (USGS, Certificate of Analysis) (Table J in S1 Table).

## *Element concentrations - results*

Calcium concentrations range from 31.1 to 32.5 wt % (Table K in S1 Table). Magnesium concentrations range from 0.04823 to 0.1163 wt %, Ti concentrations from 0.055 to 1.395 ppm, Mn concentrations from 1.40 to 479.01 ppm, Sr concentrations from 42.2 to 189.7 ppm, and Ba concentrations from 14.08 to 60.07 ppm (Table K in S1 Table). Comparing trace element concentrations to modern marsupial enamel (S6 Fig), Mg concentrations in *Protemnodon* enamel are similar to values previously measured in modern wombats [42]. WIGL8544, WIGL8549 and WIGL8550 have similar Mn concentrations to those of modern wombats. In contrast, Mn concentrations are significantly higher in both WIGL8546 (Welch Two Sample t-test, p < 0.001) and WIGL8547 (Welch Two Sample t-test, p < 0.001) when compared to modern wombats. For Sr, WIGL8544, WIGL8546 and WIGL8547 have similar concentrations to modern wombats. However, Sr concentrations in WIGL8549 (Welch Two Sample t-test, p < 0.001) and WIGL8550 (Welch Two Sample t-test, p < 0.001). Ba concentrations in all fossil specimens are significantly enriched when compared to the modern wombat enamel (Analysis of variance (Anova), p < 0.001).

Rare earth element concentrations range from 0.58 to 97.89 ppm (Table L in S1 Table). REE concentrations in WIGL8544, WIGL8549 and WIGL8550 are significantly lower than values observed in modern wombats [42](S7 Fig). In contrast, WIGL8546 and WIGL8547 have REE concentrations similar to values observed in modern wombats (S7 Fig).

For WIGL8544, WIGL8545, WIGL8549 and WIGL8550, REE concentrations do not significantly vary across individual ablation transects (S8 Fig). In WIGL8546, analyses undertaken near the enamel dentine junction (EDJ) exhibit relatively low REE concentrations, with light REE concentrations < 0.001 ppm. REE concentrations are noticeably higher towards the enamel’s outer surface with most elements exceeding 0.01 ppm at the enamel surface, to a depth of around 250 μm (S9 Fig). In WIGL8547, heavy REE concentrations appear most elevated in two transects adjacent to visible physical damage and discolouration of enamel (S10 Fig).

## *Diagenetic alteration*

Overall, *Protemnodon* specimens show REE concentrations that are similar or lower than those observed in modern wombats (S7 Fig) [42]. Samples WIGL8544, 8549 and 8550 also show Mn concentrations similar to those in modern wombats (S6 Fig) suggesting minimal diagenetic alteration [43, 44]. Contrastingly, samples WIGL8546 and WIGL8547 show higher Mn concentrations than in modern wombats (S6 Fig) and higher REE concentrations than in other fossil specimens (S7 Fig). In these two samples, REE enrichment is variable across the tooth. In WIGL8546, elevated REE concentrations occur towards the outer surfaces of enamel (S9 Fig). These results are somewhat expected as the outer layer of enamel is more susceptible to diagenesis [45, 46]. In WIGL8547, REE concentrations appear most elevated adjacent to physical damage and/or discolouration of enamel (S10 Fig). This diagenetic alteration is also somewhat expected as previous studies have shown that physical damage to enamel has the potential to facilitate fossil diagenesis, providing space for the precipitation and infilling of secondary calcium apatite [46-50]. Ultimately, these results suggest that overall *Protemnodon* enamel is relatively resistant to diagenetic alteration, though physical damage, and contact with diagenetic mediums have the potential to facilitate some degree of localised diagenesis.

For most specimens, Sr isotope values do not exhibit any correlations with indices of diagenesis such as Mn or REE concentrations, suggesting overall, ^87^Sr/^86^Sr ratios have not been diagenetically altered. For WIGL8546, localised elevated REE concentrations are correlated with shifts in ^87^Sr/^86^Sr towards Sr isotope ratios similar to that in the local Mount Etna Beds limestone (S11 Fig) [51]. Shifts in ^87^Sr/^86^Sr values towards those in Mount Etna Beds limestone are also observed in all analyses of dentine where high REE concentrations also indicate diagenesis. Dentine’s higher porosity makes it more prone to diagenetic alteration when compared to enamel [48, 52-56], thus its Sr is commonly replaced with that of the surrounding sediment [44, 57, 58]. Where enamel appears to have experienced localised diagenetic alteration in our samples, strontium isotope data were excluded from further discussion, to ensure all ^87^Sr/^86^Sr ratios reflect biologically accumulated strontium and therefore a viable proxy for estimating foraging range.

## Bio-available Strontium from local vegetation

## *Bio-available Sr – methods*

When considering Sr isotopes as a proxy for foraging range, prior studies suggest sampling of exchangeable soil Sr [59, 60], vegetation [61, 62], and mineralized tissue of extant localised species [62, 63] can be used to encapsulate localised variation in ^87^Sr/^86^Sr isotope ratios, and provide more precise estimates of foraging across more refined spatial scales [63].

Applying this to the Mt Etna Caves region, 24 plant samples were collected representing 11 distinct geological features from the 1:100K surface geology dataset (Table F in S1 Table). Vegetation sampling covered geological variation across a 500 km^2^ area around the Mt Etna Caves fossil deposits (See extent Fig 1). To minimise anthropogenic impacts on bioavailable strontium, areas with significant human modification, such as farmland and urban centres, were avoided to prevent contamination from pesticides, fertilisers, and other non-biological strontium sources [64, 65]. All necessary permits were obtained for the described study, which complied with all relevant regulations. Work was undertaken with the permission of Queensland Parks and Wildlife Service (Permit to Take, Use, Keep or Interfere P-PTUKI-100563635, Permit to Collect P-PTC-100563640).

Plant samples were prepared for analysis in a Class 10 Cleanroom, at the WIGL, University of Wollongong. Samples were allowed to dry naturally and then manually ground before being ashed at 550 ºC. Following this, 50 mg of ashed material was digested in a microwave system with 2 mL of 15 M HNO_3_. Samples were then redissolved in 2 M HNO3 before ion exchange chromatography. Strontium (Sr) was isolated from the sample matrix through automated low-pressure chromatography, using a prepFAST-MC™ equipped with a 1 mL Sr-Ca specific resin column [66-71]. The Sr elution was evaporated to dryness before being redissolved in 0.3 M HNO_3_ prior to isotopic analysis.

Strontium isotopic ratios were measured at WIGL using a Thermo-Fisher Neptune Plus MC-ICP-MS equipped with jet sample and X skimmer cones. Samples were introduced using cyclonic spray chamber equipped with an ESI Apex-St PFA MicroFlow nebuliser. Prior to analytical sessions, the instrument was tuned with a 20 ppb Sr solution of NIST SRM 987 standard, maximising sensitivity for ^88^Sr. Masses for ^85^Rb, ^86^Sr, ^87^Sr and ^88^Sr were collected on Faraday cups. Instrumental biases were corrected using the measured ^88^Sr/^86^Sr isotope ratio, and ^85^Rb was measured for isobaric interference corrections of ^87^Rb on ^87^Sr.

Accuracy was assessed with repeated analyses of NIST SRM987 throughout analytical sessions. The mean measured ^87^Sr/^87^Sr isotope ratio for SRM987 (0.710253 ± 0.000004 (2SE), n = 32) was within error of published values in Weis et al. [72] (0.710252 ± 0.000013 (2SE)). Procedural accuracy was assessed by processing an apple leaf NIST SRM1515 standard. The mean ^87^Sr/^86^Sr isotope ratio for SRM1515 (0.71389 ± 0.00007; 2SE, n = 2) was within error of the published value in Liu et al. [73] (0.71398 ± 0.00004 (2SE)). Total procedure blanks ranged between 0.005 and 0.327 ng Sr (*n* = 6). Measured bio-available ^87^Sr/^86^Sr isotopic ratios in local vegetation are reported in Table F from S1 Table.

**References**

1. Hocknull SA. Ecological succession during the late Cainozoic of central eastern Queensland: extinction of a diverse rainforest community. Memoirs of the Queensland Museum. 2005;51(1):39-122.

2. Hocknull SA, Zhao J-x, Feng Y-x, Webb GE. Responses of Quaternary rainforest vertebrates to climate change in Australia. Earth and Planetary Science Letters. 2007;264(1-2):317-31.

3. Aitken MJ. Introduction to optical dating: the dating of Quaternary sediments by the use of photon-stimulated luminescence: Clarendon Press; 1998.

4. Arnold LJ, Demuro M, Power R, Duval M, Guilarte V, Weij R, et al. Examining sediment infill dynamics at Naracoorte cave megafauna sites using multiple luminescence dating signals. Quaternary Geochronology. 2022;70:101301.

5. Arnold LJ, Demuro M, Spooner NA, Prideaux GJ, McDowell MC, Camens AB, et al. Single-grain TT-OSL bleaching characteristics: Insights from modern analogues and OSL dating comparisons. Quaternary Geochronology. 2019;49:45-51.

6. Demuro M, Arnold LJ, González‐Urquijo J, Lazuen T, Frochoso M. Chronological constraint of Neanderthal cultural and environmental changes in southwestern Europe: MIS 5–MIS 3 dating of the Axlor site (Biscay, Spain). Journal of Quaternary Science. 2023.

7. Demuro M, Arnold LJ, Parés JM, Sala R. Extended-range luminescence chronologies suggest potentially complex bone accumulation histories at the Early-to-Middle Pleistocene palaeontological site of Huéscar-1 (Guadix-Baza basin, Spain). Quaternary International. 2015;389:191-212.

8. Duller G. Distinguishing quartz and feldspar in single grain luminescence measurements. Radiation measurements. 2003;37(2):161-5.

9. Galbraith R. A note on the variance of a background-corrected OSL count. Ancient TL. 2002;20(2):49-51.

10. Jacobs Z, Duller GA, Wintle AG. Interpretation of single grain De distributions and calculation of De. Radiation Measurements. 2006;41(3):264-77.

11. Duller GA. Assessing the error on equivalent dose estimates derived from single aliquot regenerative dose measurements. Ancient TL. 2007;25(1):15-24.

12. Arnold LJ, Duval M, Falguères C, Bahain J-J, Demuro M. Portable gamma spectrometry with cerium-doped lanthanum bromide scintillators: Suitability assessments for luminescence and electron spin resonance dating applications. Radiation Measurements. 2012;47(1):6-18.

13. Duval M, Arnold LJ. Field gamma dose-rate assessment in natural sedimentary contexts using LaBr3 (Ce) and NaI (Tl) probes: A comparison between the “threshold” and “windows” techniques. Applied Radiation and Isotopes. 2013;74:36-45.

14. Bøtter-Jensen L, Mejdahl V. Assessment of beta dose-rate using a GM multicounter system. International Journal of Radiation Applications and Instrumentation Part D Nuclear Tracks and Radiation Measurements. 1988;14(1-2):187-91.

15. Potts PJ, Thompson M, Chenery SR, Webb PC, Kasper HU. GeoPT13-An international proficiency test for analytical geochemistry laboratories-report on round 13/July 2003 (Köln Loess). International Association of Geoanalysts. 2003:1-38.

16. Leslie C. Analysing environmental radioactivity in soils and sediments using high-purity germanium gamma detectors at CSIRO Land and Water: Procedures and Protocols: Citeseer; 2009.

17. Prescott JR, Hutton JT. Cosmic ray contributions to dose rates for luminescence and ESR dating: large depths and long-term time variations. Radiation measurements. 1994;23(2-3):497-500.

18. Allkofer OC. Introduction to cosmic radiation. NASA STI/Recon Technical Report A. 1975;75:46929.

19. Smith M, Prescott JR, Head M. Comparison of 14C and luminescence chronologies at Puritjarra rock shelter, central Australia. Quaternary science reviews. 1997;16(3-5):299-320.

20. Mejdahl V. Internal radioactivity in quartz and feldspar grains. Ancient TL. 1987;5(2):10-7.

21. Bowler JM, Johnston H, Olley JM, Prescott JR, Roberts RG, Shawcross W, et al. New ages for human occupation and climatic change at Lake Mungo, Australia. Nature. 2003;421(6925):837-40.

22. Jacobs Z, Duller GA, Wintle AG, Henshilwood CS. Extending the chronology of deposits at Blombos Cave, South Africa, back to 140 ka using optical dating of single and multiple grains of quartz. Journal of Human Evolution. 2006;51(3):255-73.

23. Pawley SM, Bailey RM, Rose J, Moorlock BS, Hamblin RJ, Booth SJ, et al. Age limits on Middle Pleistocene glacial sediments from OSL dating, north Norfolk, UK. Quaternary Science Reviews. 2008;27(13-14):1363-77.

24. Lewis RJ, Tibby J, Arnold LJ, Barr C, Marshall J, McGregor G, et al. Insights into subtropical Australian aridity from Welsby Lagoon, north Stradbroke Island, over the past 80,000 years. Quaternary Science Reviews. 2020;234:106262.

25. Rees-Jones J. Optical dating of young sediments using fine-grain quartz. Ancient TL. 1995;13(2):9-14.

26. Rees‐Jones J, Tite M. Optical dating results for British archaeological sediments. Archaeometry. 1997;39(1):177-87.

27. Readhead M. Absorbed dose fraction for 87Rb β particles. Ancient TL. 2002;20(1):25-8.

28. Guérin G, Mercier N, Adamiec G. Dose-rate conversion factors: update. Ancient Tl. 2011;29(1):5-8.

29. Mejdahl V. Thermoluminescence dating: beta-dose attenuation in quartz grains. Archaeometry. 1979;21(pt. 1):61-72.

30. Brennan B. Beta doses to spherical grains. Radiation Measurements. 2003;37(4-5):299-303.

31. Aitken M. Thermoluminescence Dating Academic Press New York. 1985.

32. Readhead M. Thermoluminescence dose rate data and dating equations for the case of disequilibrium in the decay series. International Journal of Radiation Applications and Instrumentation Part D Nuclear Tracks and Radiation Measurements. 1987;13(4):197-207.

33. Arnold LJ, Demuro M. Insights into TT-OSL signal stability from single-grain analyses of known-age deposits at Atapuerca, Spain. Quaternary Geochronology. 2015;30:472-8.

34. Arnold LJ, Demuro M, Parés JM, Arsuaga JL, Aranburu A, de Castro JMB, et al. Luminescence dating and palaeomagnetic age constraint on hominins from Sima de los Huesos, Atapuerca, Spain. Journal of human evolution. 2014;67:85-107.

35. Demuro M, Arnold LJ, Aranburu A, Gómez-Olivencia A, Arsuaga J-L. Single-grain OSL dating of the Middle Palaeolithic site of Galería de las Estatuas, Atapuerca (Burgos, Spain). Quaternary Geochronology. 2019;49:254-61.

36. Demuro M, Arnold LJ, Parés JM, Pérez-González A, Ortega AI, Arsuaga JL, et al. New luminescence ages for the Galería Complex archaeological site: resolving chronological uncertainties on the acheulean record of the Sierra de Atapuerca, Northern Spain. PLoS One. 2014;9(10):e110169.

37. Zilhao J, Angelucci DE, Arnold LJ, Demuro M, Hoffmann DL, Pike AW. A revised, last interglacial chronology for the Middle Palaeolithic sequence of Gruta da Oliveira (Almonda karst system, Torres Novas, Portugal). Quaternary Science Reviews. 2021;258:106885.

38. Zilhão J, Angelucci DE, Igreja MA, Arnold LJ, Badal E, Callapez P, et al. Last Interglacial Iberian Neandertals as fisher-hunter-gatherers. Science. 2020;367(6485):eaaz7943.

39. Slimak L, Zanolli C, Higham T, Frouin M, Schwenninger J-L, Arnold LJ, et al. Modern human incursion into Neanderthal territories 54,000 years ago at Mandrin, France. Science advances. 2022;8(6):eabj9496.

40. Fusco DA, Arnold LJ, Gully GA, Levchenko VA, Jacobsen GE, Prideaux GJ. Revisiting the late Quaternary fossiliferous infills of Cathedral Cave, Wellington Caves (central eastern New South Wales, Australia). Journal of Quaternary Science. 2023.

41. Paton C, Hellstrom J, Paul B, Woodhead J, Hergt J. Iolite: Freeware for the visualisation and processing of mass spectrometric data. Journal of Analytical Atomic Spectrometry. 2011;26(12):2508-18.

42. Koutamanis D, McCurry M, Tacail T, Dosseto A. Reconstructing Pleistocene Australian herbivore megafauna diet using calcium and strontium isotopes. Royal Society Open Science. 2023;10(11):230991.

43. Reynard B, Balter V. Trace elements and their isotopes in bones and teeth: Diet, environments, diagenesis, and dating of archeological and paleontological samples. Palaeogeography, Palaeoclimatology, Palaeoecology. 2014;416:4-16.

44. Xingyue W, Chengshan W, Huang C, Song B, Zhang Q. A novel method to assess the effect of diagenesis on fossil teeth: Rare earth element signatures. Journal of Rare Earths. 2011;29(7):710-5.

45. Abdallah M-N, Eimar H, Bassett DC, Schnabel M, Ciobanu O, Nelea V, et al. Diagenesis-inspired reaction of magnesium ions with surface enamel mineral modifies properties of human teeth. Acta biomaterialia. 2016;37:174-83.

46. Jacques L, Ogle N, Moussa I, Kalin R, Vignaud P, Brunet M, et al. Implications of diagenesis for the isotopic analysis of Upper Miocene large mammalian herbivore tooth enamel from Chad. Palaeogeography, Palaeoclimatology, Palaeoecology. 2008;266(3-4):200-10.

47. Hedges RE. Bone diagenesis: an overview of processes. Archaeometry. 2002;44(3):319-28.

48. Kendall C, Eriksen AMH, Kontopoulos I, Collins MJ, Turner-Walker G. Diagenesis of archaeological bone and tooth. Palaeogeography, palaeoclimatology, palaeoecology. 2018;491:21-37.

49. Austin C, Smith TM, Bradman A, Hinde K, Joannes-Boyau R, Bishop D, et al. Barium distributions in teeth reveal early-life dietary transitions in primates. Nature. 2013;498(7453):216-9.

50. Brumfitt I, Chinsamy A, Compton J. Depositional environment and bone diagenesis of the Mio/Pliocene Langebaanweg bonebed, South Africa. South African Journal of Geology. 2013;116(2):241-58.

51. Deer LN. Limestone and speleothem trace element geochemistry as tools for palaeoclimatic reconstruction, Mount Etna region, central-coastal Queensland: Queensland University of Technology; 2011.

52. Budd P, Montgomery J, Barreiro B, Thomas RG. Differential diagenesis of strontium in archaeological human dental tissues. Applied Geochemistry. 2000;15(5):687-94. doi: <https://doi.org/10.1016/S0883-2927(99)00069-4>.

53. Copeland SR, Sponheimer M, Lee-Thorp JA, le Roux PJ, de Ruiter DJ, Richards MP. Strontium isotope ratios in fossil teeth from South Africa: assessing laser ablation MC-ICP-MS analysis and the extent of diagenesis. Journal of Archaeological Science. 2010;37(7):1437-46. doi: <https://doi.org/10.1016/j.jas.2010.01.003>.

54. de Sousa DV, Eltink E, Oliveira RAP, Félix JF, Guimarães LdM. Diagenetic processes in Quaternary fossil bones from tropical limestone caves. Scientific reports. 2020;10(1):1-16.

55. Kohn MJ, Schoeninger MJ, Barker WW. Altered states: effects of diagenesis on fossil tooth chemistry. Geochimica et cosmochimica acta. 1999;63(18):2737-47.

56. Pye K. Isotope and trace element analysis of human teeth and bones for forensic purposes. Geological Society, London, Special Publications. 2004;232(1):215-36.

57. Tütken T, Vennemann TW. Fossil bones and teeth: preservation or alteration of biogenic compositions? Palaeogeography, Palaeoclimatology, Palaeoecology. 2011;1(310):1-8.

58. Burton JH, Price TD. Seeking the local 87Sr/86Sr ratio to determine geographic origins of humans. Archaeological chemistry VIII: ACS Publications; 2013. p. 309-20.

59. de Caritat P, Dosseto A, Dux F. A strontium isoscape of northern Australia. Earth System Science Data Discussions. 2023:1-32.

60. de Caritat P, Dosseto A, Dux F. A strontium isoscape of inland southeastern Australia. Earth System Science Data. 2022;14(9):4271-86.

61. Hoppe KA, Koch PL, Carlson RW, Webb SD. Tracking mammoths and mastodons: Reconstruction of migratory behavior using strontium isotope ratios. Geology. 1999;27(5):439-42. doi: 10.1130/0091-7613(1999)027<0439:TMAMRO>2.3.CO;2.

62. Adams S, Grün R, McGahan D, Zhao J-X, Feng Y, Nguyen A, et al. A strontium isoscape of north-east Australia for human provenance and repatriation. Geoarchaeology. 2019. doi: 10.1002/gea.21728.

63. Funck J, Bataille C, Rasic J, Wooller M. A bio‐available strontium isoscape for eastern Beringia: a tool for tracking landscape use of Pleistocene megafauna. Journal of Quaternary Science. 2021;36(1):76-90.

64. Snoeck C, Ryan S, Pouncett J, Pellegrini M, Claeys P, Wainwright AN, et al. Towards a biologically available strontium isotope baseline for Ireland. Science of The Total Environment. 2020;712:136248.

65. Maurer A-F, Galer SJ, Knipper C, Beierlein L, Nunn EV, Peters D, et al. Bioavailable 87Sr/86Sr in different environmental samples—Effects of anthropogenic contamination and implications for isoscapes in past migration studies. Science of the Total Environment. 2012;433:216-29.

66. Balter V, Martin JE, Tacail T, Suan G, Renaud S, Girard C. Calcium stable isotopes place Devonian conodonts as first level consumers. Geochemical Perspectives Letters. 2019:36-9. doi: 10.7185/geochemlet.1912.

67. Martin JE, Tacail T, Adnet S, Girard C, Balter V. Calcium isotopes reveal the trophic position of extant and fossil elasmobranchs. Chemical Geology. 2015;415:118-25. doi: 10.1016/j.chemgeo.2015.09.011.

68. Martin JE, Tacail T, Braga J, Cerling TE, Balter V. Calcium isotopic ecology of Turkana Basin hominins. Nat Commun. 2020;11(1):3587. Epub 2020/07/19. doi: 10.1038/s41467-020-17427-7. PubMed PMID: 32681008.

69. Martin JE, Tacail T, Cerling TE, Balter V. Calcium isotopes in enamel of modern and Plio-Pleistocene East African mammals. Earth and Planetary Science Letters. 2018;503:227-35. doi: 10.1016/j.epsl.2018.09.026.

70. Tacail T, Albalat E, Télouk P, Balter V. A simplified protocol for measurement of Ca isotopes in biological samples. Journal of Analytical Atomic Spectrometry. 2014;29(3):529-35.

71. Koutamanis D, Roberts GL, Dosseto A. Inter- and intra-individual variability of calcium and strontium isotopes in modern Tasmanian wombats. Palaeogeography, Palaeoclimatology, Palaeoecology. 2021;574. doi: 10.1016/j.palaeo.2021.110435.

72. Weis D, Kieffer B, Maerschalk C, Barling J, De Jong J, Williams GA, et al. High‐precision isotopic characterization of USGS reference materials by TIMS and MC‐ICP‐MS. Geochemistry, Geophysics, Geosystems. 2006;7(8).

73. Liu H-C, Chung C-H, You C-F, Chiang Y-H. Determination of Sr/Sr and δSr ratios in plant materials using MC-ICP-MS. Analytical & Bioanalytical Chemistry. 2016;408(2).
