# Supplementary figures and images for: Megafauna mobility: Assessing the foraging range of an extinct macropodid from central eastern Queensland, Australia
Source: PLoS One. 2025 Apr 23;20(4):e0319712. doi: 10.1371/journal.pone.0319712 (PMC12017834; doi:10.1371/journal.pone.0319712)

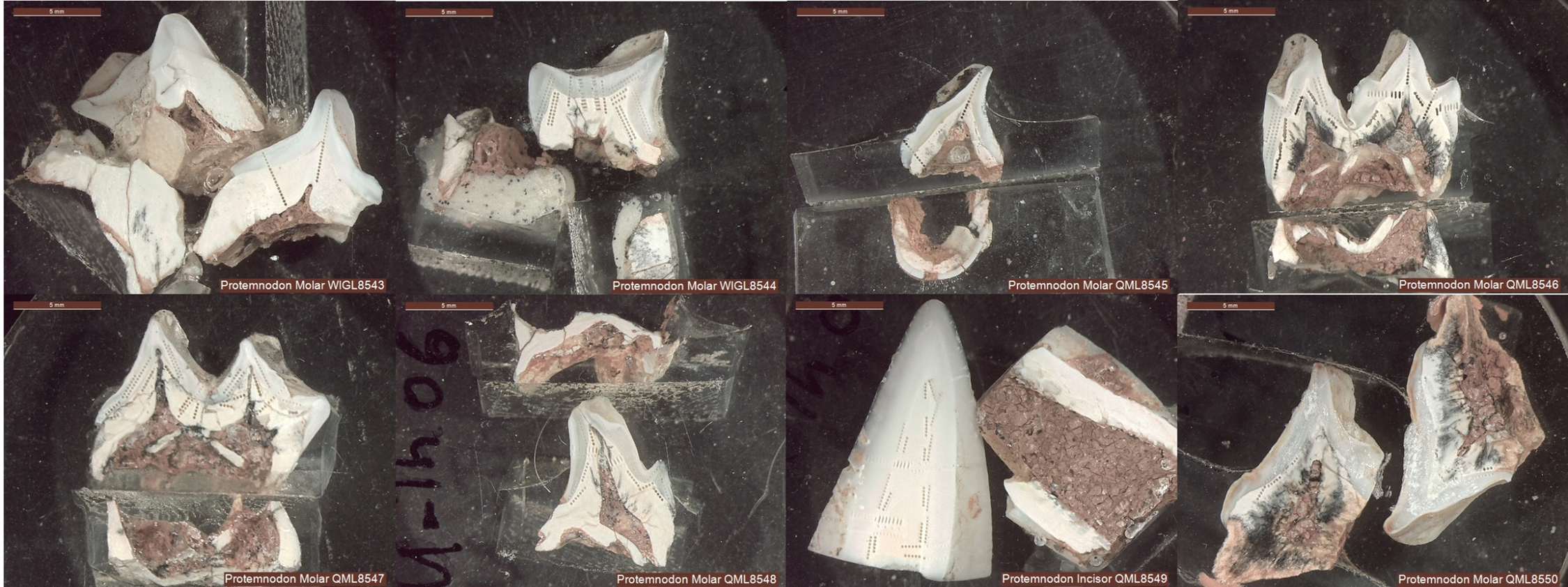

Supplement: S1 Fig — Samples QML1311H-WIGL8543 to QML1312-WIGL8554. Strontium transects can be identified by a series of large depressions running along the enamel/dentine. (TIF) [file pone.0319712.s003.tif]

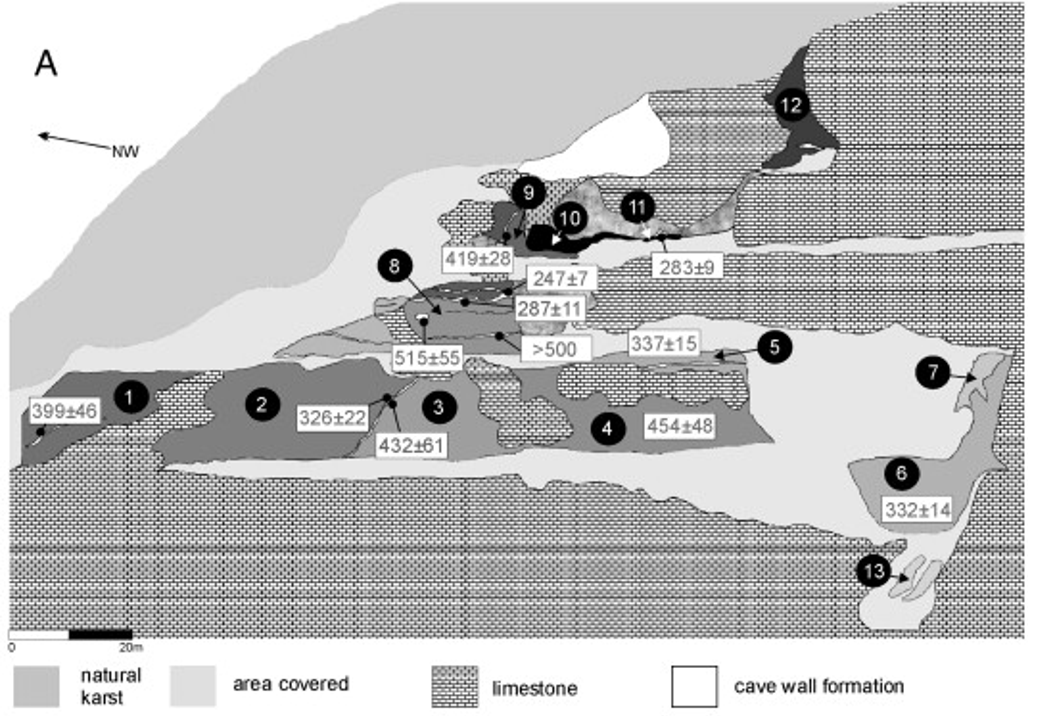

Supplement: S2 Fig — Exposed fossil deposits at Mt Etna Limestone Mine, western benches. 1–5 QML1311; 1. A/B, 2. C/D, 3. F, 4. H, 5. J. 6–7 QML1384; 6. LU, 7. UU. 8. QML1310 Unit 2, 9. QML1383 A, 10. Open chamber to Speaking Tub Cave System, 11. QML1313, 12. Bench 0 (A/B), 13. QML1385. Reprinted with permission from Hocknull et al. [33] © 2007 Elsevier B.V. All rights reserved. (TIF) [file pone.0319712.s004.tif]

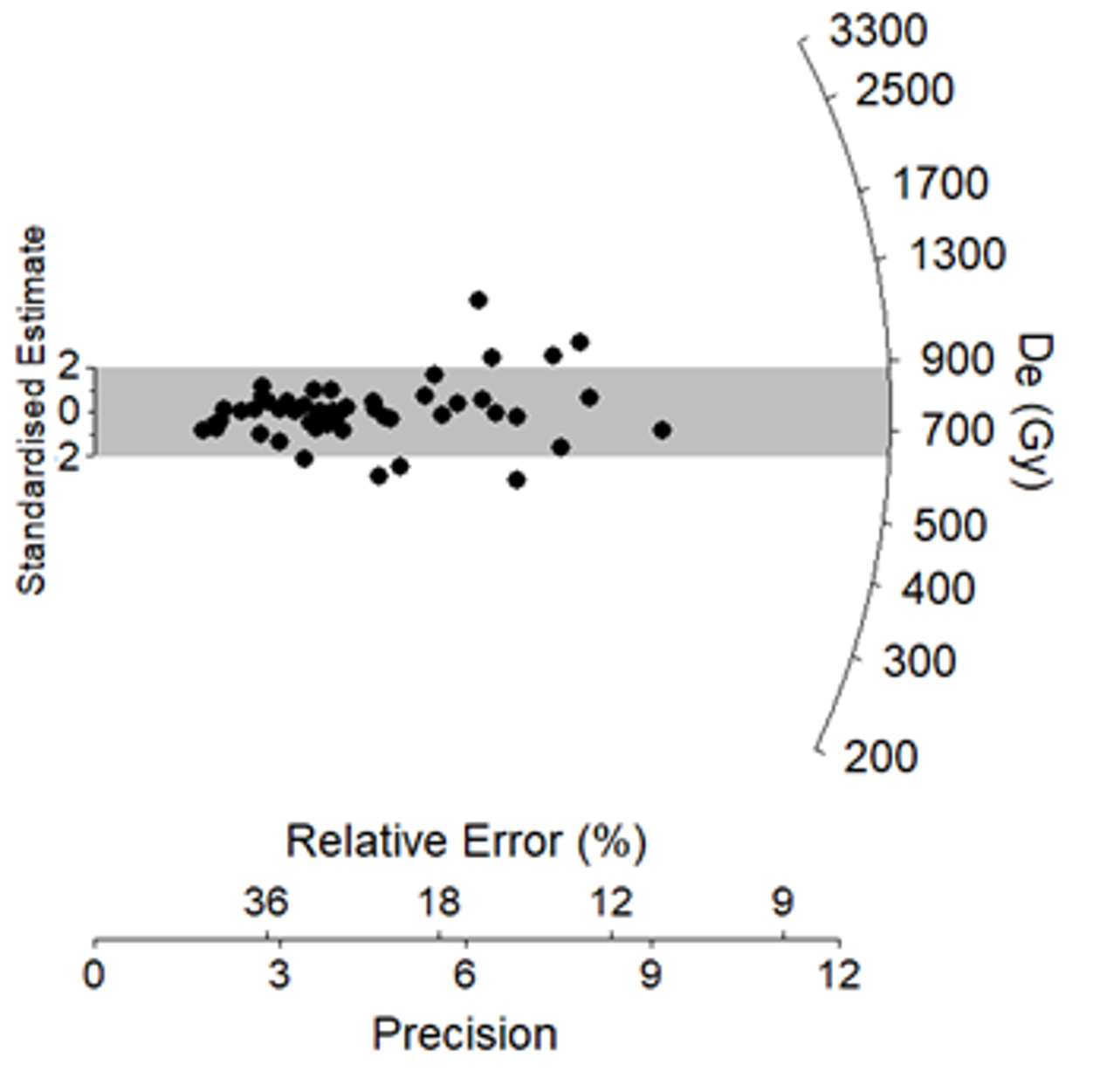

Supplement: S3 Fig — Radial plot showing the dose-recovery test (natural + dosed) TT-OSL De values obtained for sample MTE17-4 after applying the SAR quality assurance criteria. The grey band is centred on the weighted mean De value of the unbleached and dosed grains in the dose recovery test, calculated using the central age model (CAM) [86]. (TIF) [file pone.0319712.s005.tif]

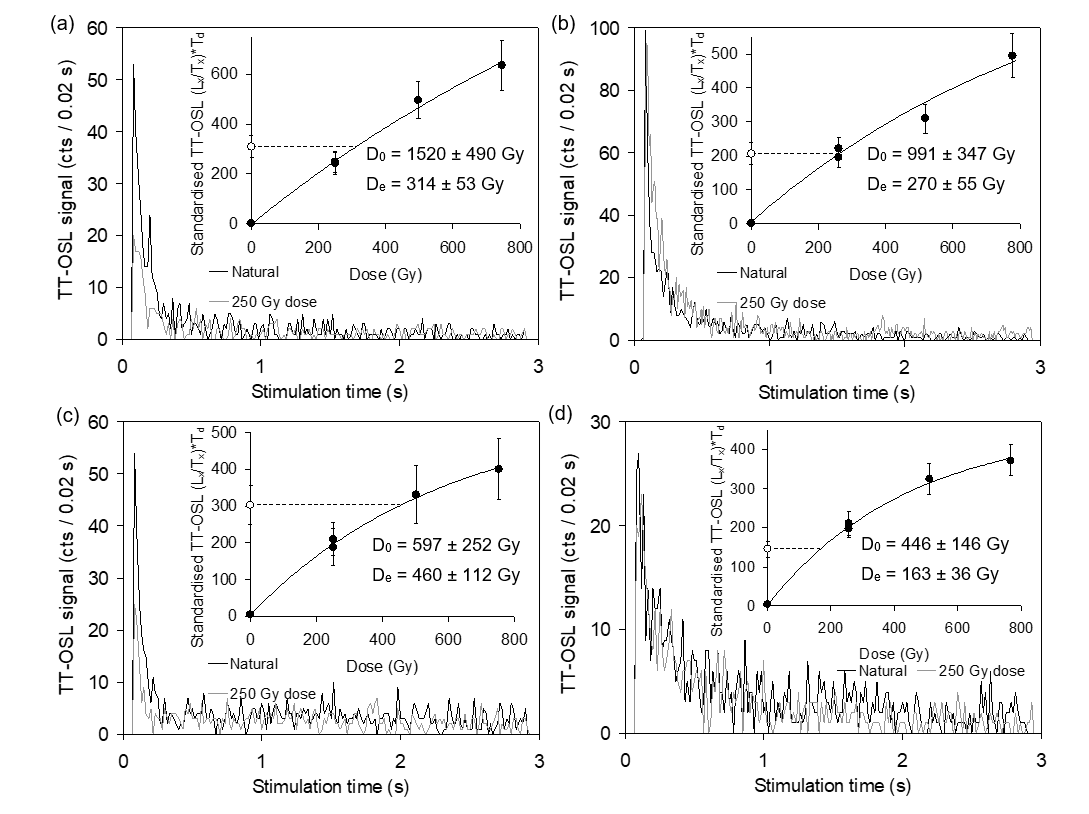

Supplement: S4 Fig — In the insets, the open circle denotes the sensitivity-corrected natural signal, and filled circles denote the sensitivity-corrected regenerative dose signals. The D0 value characterises the rate of signal saturation with respect to administered dose and equates to the dose value for which the saturating exponential dose-response curve slope is 1/e (or ~ 0.37) of its initial value. (TIF) [file pone.0319712.s006.tif]

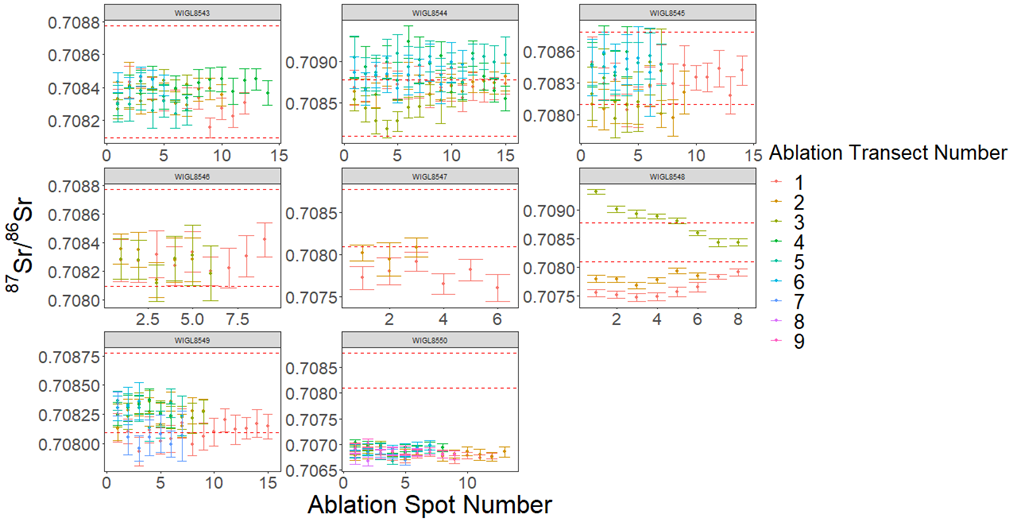

Supplement: S5 Fig — (TIF) [file pone.0319712.s007.tif]

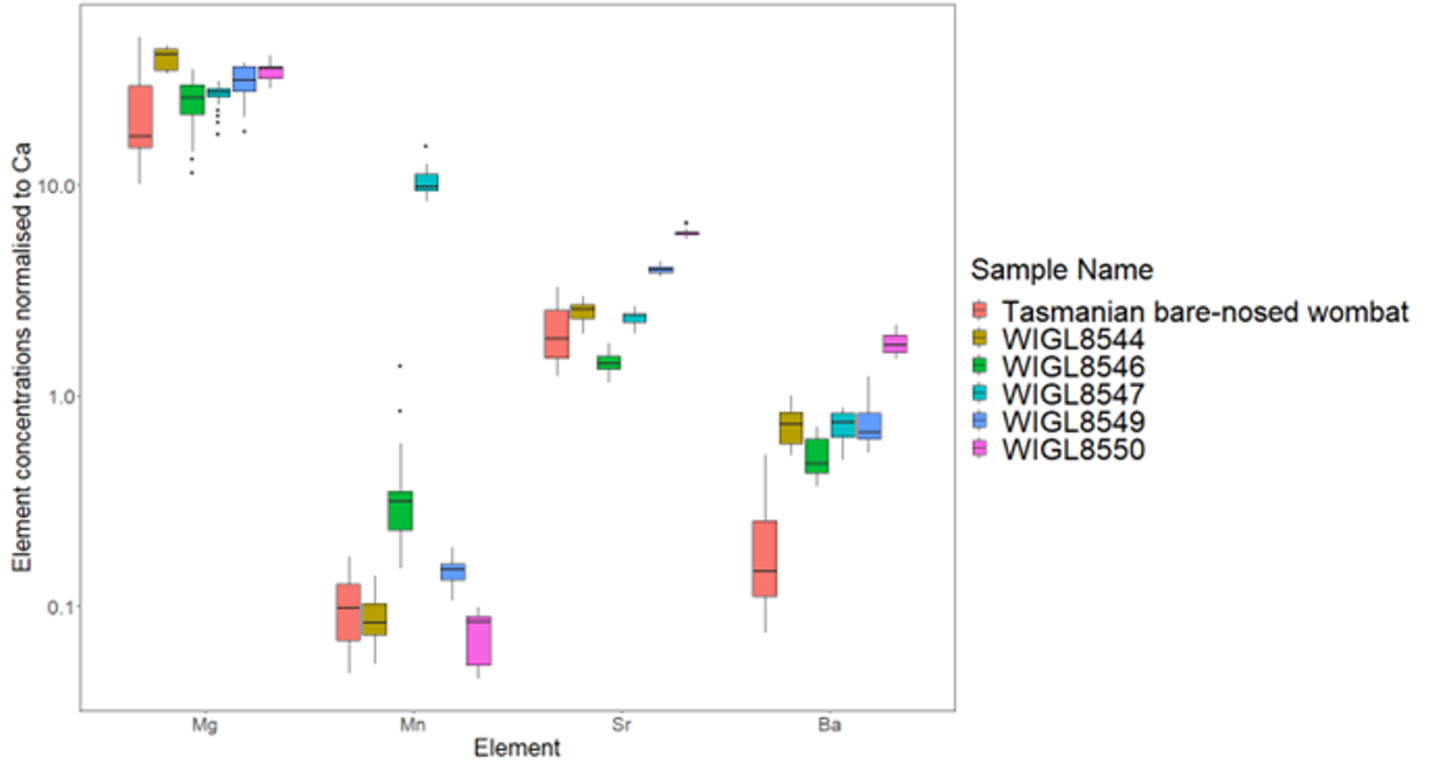

Supplement: S6 Fig — Data has been normalised to Ca, and compared to modern Tasmanian bare-nosed wombats reported in Koutamanis et al. [7]. (TIF) [file pone.0319712.s008.tif]

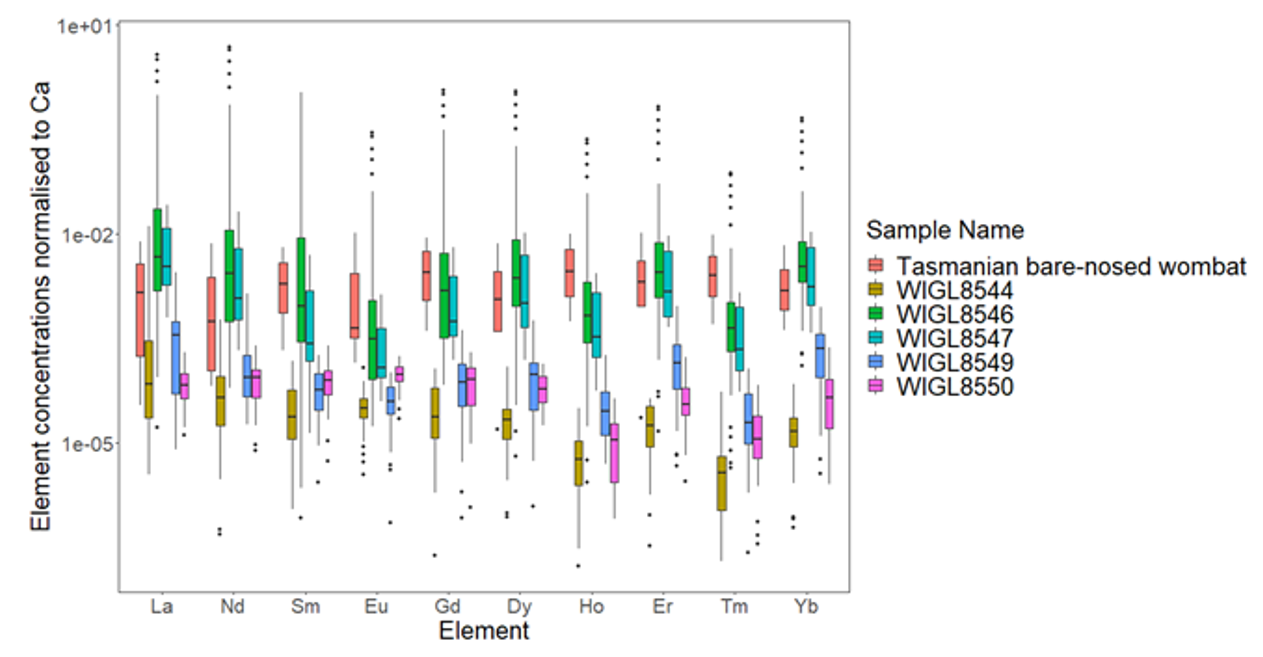

Supplement: S7 Fig — Results are compared to modern Tasmanian bare-nosed wombats reported in Koutamanis et al. [7]. (TIF) [file pone.0319712.s009.tif]

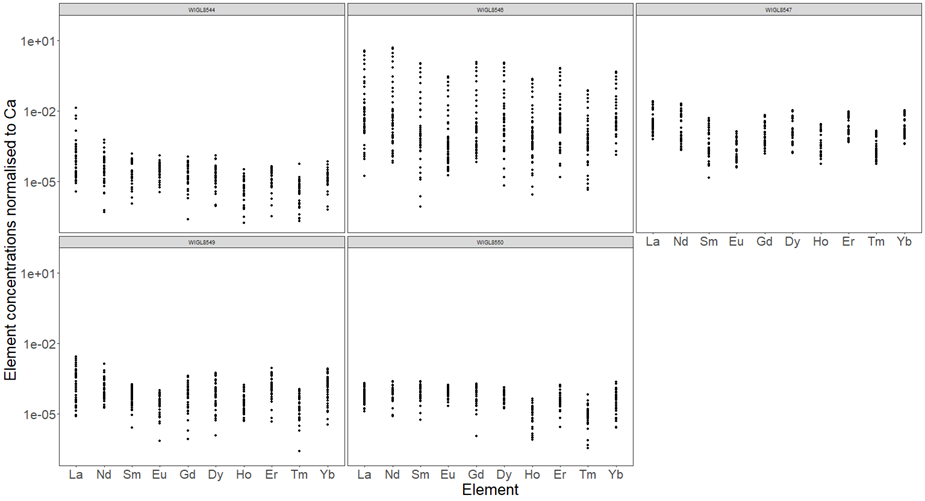

Supplement: S8 Fig — (TIF) [file pone.0319712.s010.tif]

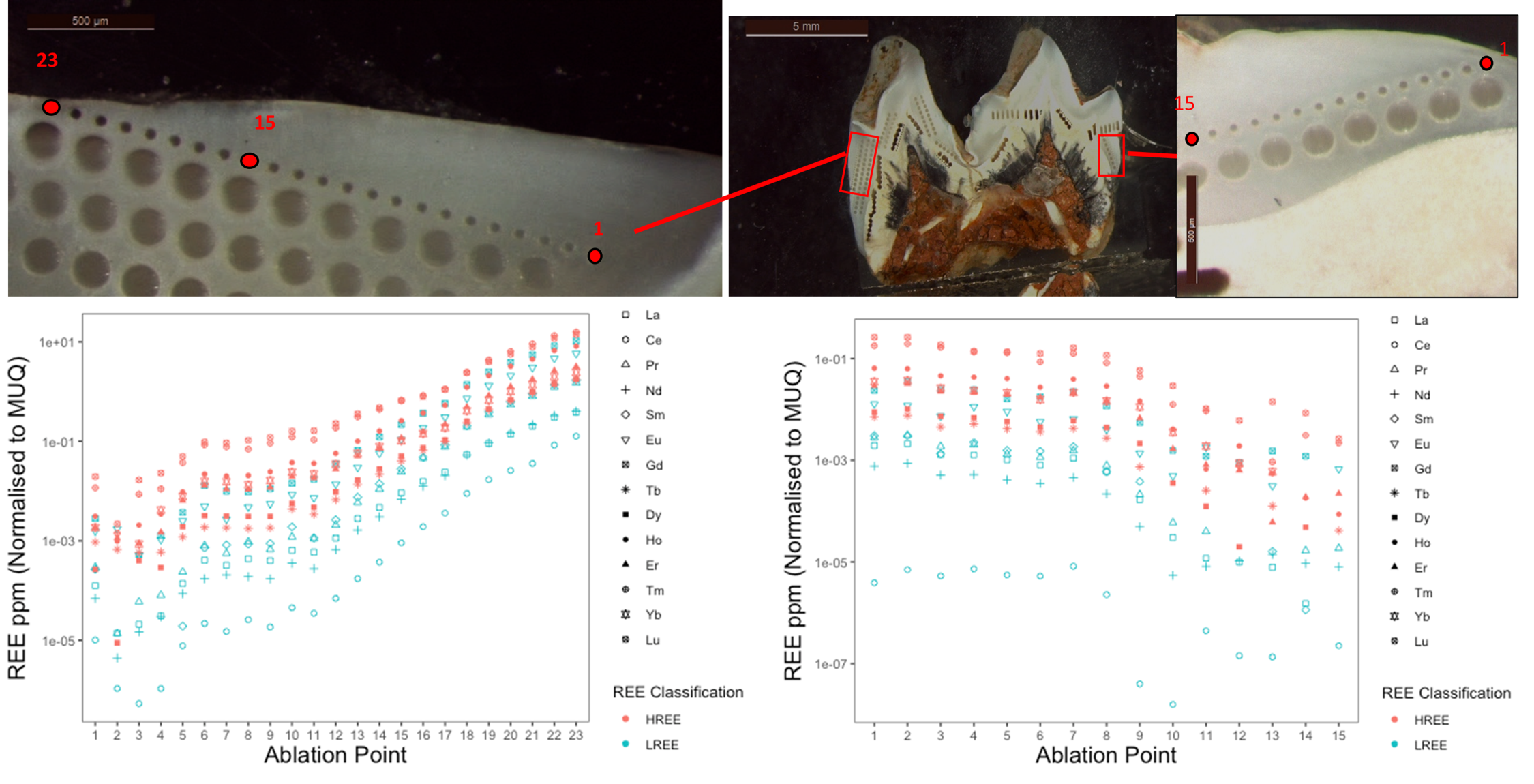

Supplement: S9 Fig — Transect 1 (Left) runs from the crown towards the base of tooth and enamel edge (1-23). Transect 2 (right) runs from the base of the enamel towards the crown (1-15). (TIF) [file pone.0319712.s011.tif]

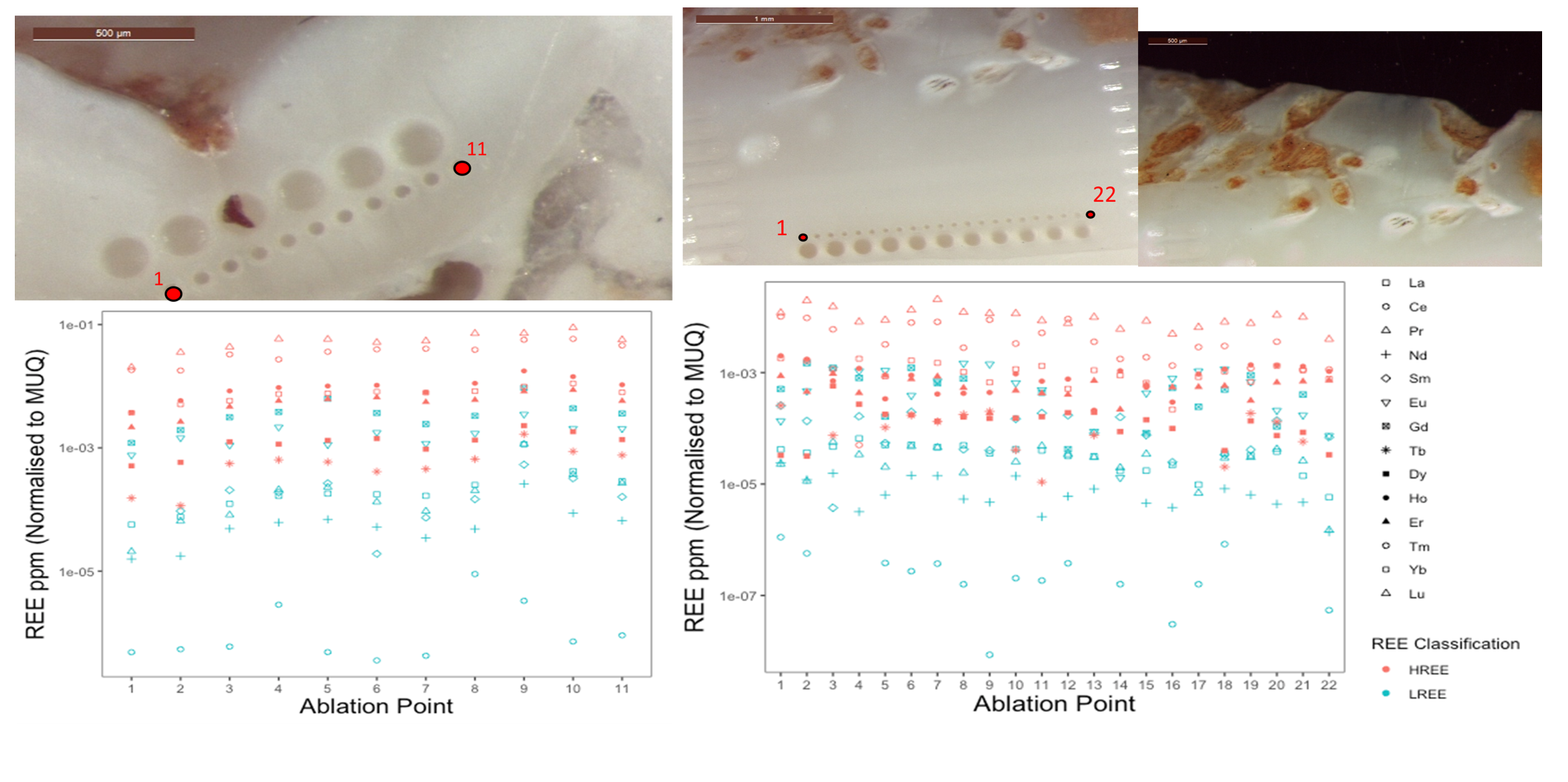

Supplement: S10 Fig — Transect 1 (Left) along the enamel boundary, adjacent to discolouration (1-11). Transect 2 (right) runs alongside to damaged and discoloured enamel (1-15). (TIF) [file pone.0319712.s012.tif]

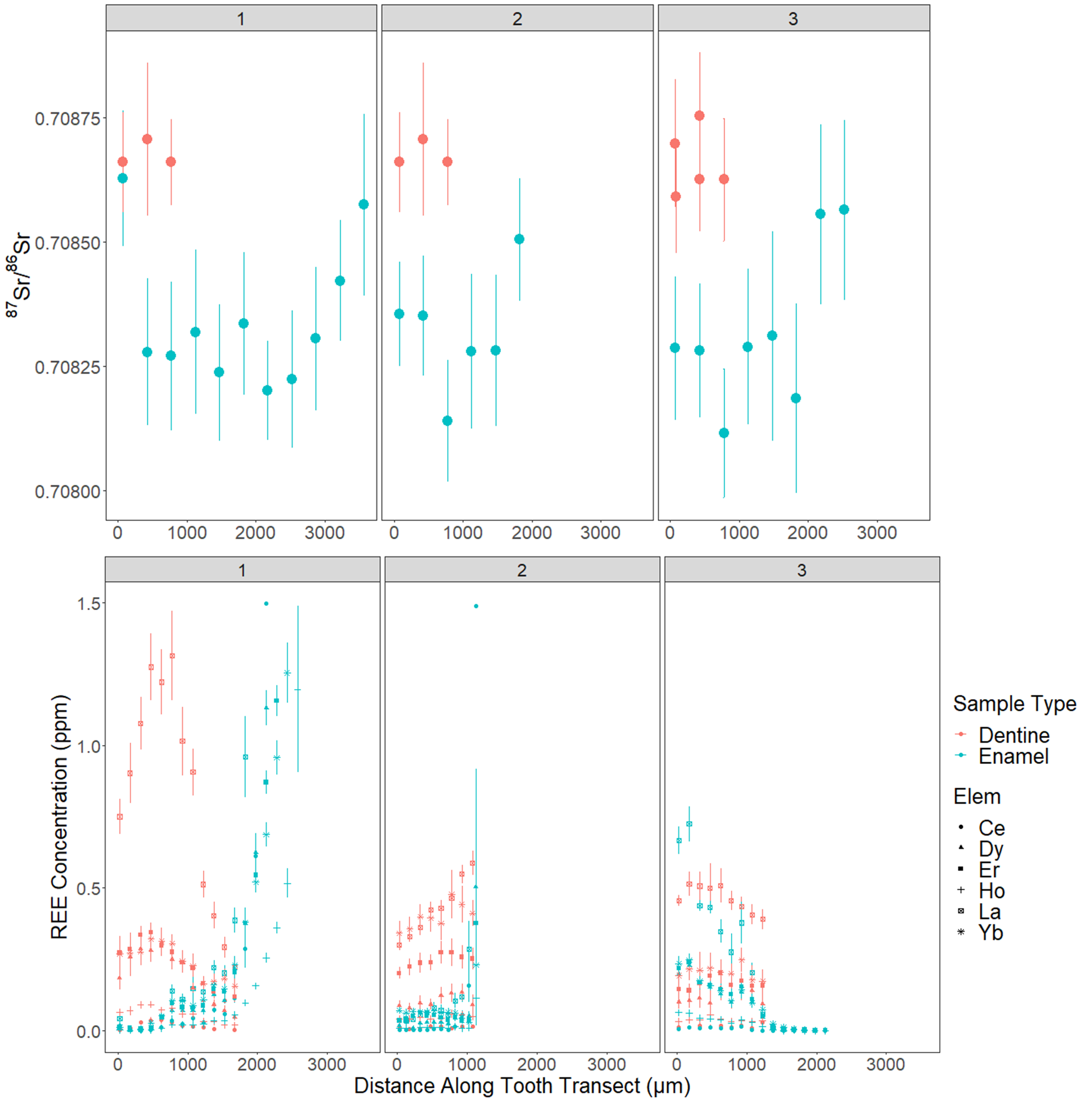

Supplement: S11 Fig — (TIF) [file pone.0319712.s013.tif]
