# Supplementary material for: Megafauna mobility: Assessing the foraging range of an extinct macropodid from central eastern Queensland, Australia
Source: PLoS One. 2025 Apr 23;20(4):e0319712. doi: 10.1371/journal.pone.0319712 (PMC12017834; doi:10.1371/journal.pone.0319712)
Supplement: Attachment — Submitted filename: Response_to_Reviewers_auresp_2.docx [file pone.0319712.s016.docx]

**Response to Reviewers**

**Reviewer #2:**

 Once again, this study Is the result of original research and as far as I can discern results have not been reported elsewhere. Methods are well described and rigorously performed to a high standard. The article is intelligible and written in standard English. Data availability guidelines are also met. However, I am puzzled by seemingly contradictory narratives even though the manuscript has been significantly revised and new temporal data added.

First, the authors note that 24 local plants were sampled for Sr, in an attempt to meet the need for a background Sr dataset suggested in previous reviews. Values are reported in S1_Table, ranging from 0.705817-0.709395, a much wider range of Sr values than those seen in *Protemnodon*. These data appear to support the authors’ argument but are not discussed in the manuscript. Were these samples collected within the foraging range of *Protemnodon* based on estimates of body mass or within the narrower foraging range proposed by the authors? The plant study is mentioned in Lines 314-316 only. A more in-depth treatment of these data would be useful.

- Mention of Plant data in lines 314 onwards has been explained in more depth. Text now reads “To examine variations in Sr isotopes across geological substrates, ^87^Sr/^86^Sr isotope ratios were measured in 24 plant samples representing the 11 distinct geological features encapsulating a 275 km^2^ region around Mt Etna (Fig. 1, S1 File, Table F in S1 Table). As bio-available ^87^Sr/^86^Sr is associated with underlying geology (Adams et al., 2019, Zieliński et al., 2021), using this plant dataset, movements in *Protemnodon* could be defined as localised (constrained to the Mt Etna Beds limestone) or reflect larger scale movement to adjacent geological substrates.”
- The extent of plant sampling is now also displayed in Fig. 1 showing these 24 samples cover the 275 km^2^ area.
- Plant data is now incorporated in the discussion & Results as follows:
  - In Fig. 5. The ^87^Sr/^86^Sr isotope ratios in enamel are now compared against the mean (± 2SE) ^87^Sr/^86^Sr measured in the various geological substrates measured around Mt Etna Caves.
  - Relationships between Sr isotopes measured in enamel and surrounding geology is now discussed further in the results and discussion of the paper suggesting movements of most *Protemnodon* were constrained to the broader Mount Alma Formation, and local limestone in 4 individuals.

I am also puzzled by conclusions the authors’ reach based on S5_Fig. This figure shows that a substantial subset of measured Sr values falls outside the Mt Etna caves’ range in five of the eight teeth sampled, given that dotted lines mark the range of Sr values on Mt Etna limestone (Line 410-412). Rather than fully acknowledging this finding the authors argue that most sampling units overlap those of Mt Etna limestone attributing the bulk of the variability to a single dental sample with Sr values entirely outside this range (Line 46-418). A more conservative interpretation of reported Sr data would argue that whereas four of the individuals sampled appear to have foraged within a narrow Mt Etna home range, others foraged more widely. Given a relatively wide range of body mass estimates for this taxon (50-170kg, Lines 325-328), this finding seems reasonable. U-Th ages reported in Table 2 do not suggest that these differences are temporally patterned. Thus, variability in foraging behavior seems a parsimonious interpretation of the data.

- Using Sr data for distinct geological features (Now included in Fig. 5), we show that the foraging ranges of all individuals (excluding one) are constrained to the range of ^87^Sr/^86^Sr isotope ratios measured in the Mount Alma Formation, the broader formation that hosts the local limestone units in which the caves are formed. Therefore, we can still maintain the conclusion that most Protemnodon were highly localised constrained to just the Mount Alma Formation (See Fig. 1) whereas modelled foraging ranges based on body mass should extend into surrounding geological features.
- Moreover, four individuals show Sr composition falling within the range of values measured across Mount Alma Formation limestone, the limestone bed which houses fossil deposits.
- As we see similar ^87^Sr/^86^Sr isotope ratios in Ellrott Rhyolite (South of Mount Etna), we suggest that whilst it is plausible individuals may have resided south of Mount Etna caves.
  - However, as the Rhyolite is interspersed between geological units (Gabbro, Lakes Creek Formation etc.) with significantly lower Sr isotope composition, ^87^Sr/^86^Sr isotope ratios we suggest that Protemnodon in this ‘southern’ habitat are more likely to be characterised by a lower isotope ratio reflecting foraging across several of these substrates.
  - Moreover, while it could be argued that some environmental factor may have constrained individual foraging ranges to vegetation underlain with Rhyolite, during enamel mineralisation. Given all teeth are found in Mount Etna deposits, we argue that across a broad time frame (280 ka to > 330 ka) it is highly unlikely that all individuals were non-vagile during enamel mineralisation, but then undertook a one-way > 7 km northward journey and became a part of the fossil record.
- Ultimately we conclude, that broader sampling of ^87^Sr/^86^Sr across geological substates, and a spatial Sr isoscape are necessary to accurately model specific foraging ranges, the most probable interpretation of our data is a local origin, constrained to the Mount Alma Formation in most individuals, and further constrained to the local limestone in 4.
- For the *Protemnodon* with a significantly lower Sr isotope composition, using ^87^Sr/^86^Sr in surrounding geological features we can suggest that the individual foraged over a different substratum; potentially Alton Downs Basalt, Permian to Triassic Gabbro or alluvial deposits associated with the Fitzroy River.
  - Based on the spatial distribution of these features we can suggest that during enamel mineralisation this individual originated at least > 1 km from fossil bearing caves (See Fig 1. local units of Gabbro).

Moreover, if *Protemnodon* was foraging in a closed canopy rainforest enamel d13C values will be diagnostic of that setting, more negative than expected of a C3 open foraging environment given the recycling of atmospheric CO2. Results will either support or call into question the authors’ argument that *Protemnodon’s* foraging range was narrow and determined by climate rather than scaled to body mass. This is a straightforward analytical procedure, well established methodologically. It was suggested in a previous review but not conducted or reported here.

- We agree that carbon isotope would significantly strengthen arguments for small foraging ranges within a closed-forest habitat. However, additional analyses were beyond the scope of this study, however, we have taken the idea onboard and have plans to implement in future studies of Mt Etna.
- Despite the lack of C isotopes, arguments for a closed-forest/rainforest habitat surrounding Mount Etna Caves (> 280 ka) is not a novel idea, but one supported by a large base of pre-existing literature etc.
  - (Hocknull et al., 2007) “All of the dated sites older than ∼ 280 ka cluster together and with the New Guinean and northern Australian tropical rainforest faunas”
  - (Price et al., 2017a) suggests “The story of the Mt Etna region is somewhat similar to that of Riversleigh. The oldest fossil faunas recovered include many species that appear to have been rainforest specialists, some of which share close evolutionary links to those found today in lowland rainforests of New Guinea today”
- Therefore, we argue attributing local Sr isotopes (and small ranges) to this proposed productive forest habitat – even without C isotopes – is still a viable conclusion.
- Moreover, multiple existing studies using Sr isotopes often draw similar conclusions about environmental quality without C isotopes – See Koutamanis et al. (2023), (Radloff et al., 2010)
- In summation, C isotopes were beyond the scope of this study, we appreciate the suggestion and will incorporate them into future studies expanding on this preliminary work.

**Reviewer #3:**

The study presents novel and original research into the mobility and foraging ranges of mid-Pleistocene Mt Etna Protemnodon populations, as well as further dating constraints on two Mt Etna stratigraphic units. Overall, the paper is written succinctly, with a detailed methodology outlining how the study was conducted. Moreover, the data presented in the paper appears to support the conclusions made by the authors. Measured Sr values within the teeth appear to be similar to the bio-available strontium levels within the Mt Etna region, suggesting that the local Protemnodon had limited foraging ranges. The dates also appear to be consistent with previously published data on the sites.

**Lines 197-207:** This section is difficult to follow. I think the authors are saying that: 1) the selected teeth could be assigned to the genus *Protemnodon*, but not to a species; and 2) the dataset represents home ranges and mobility of mid-Pleistocene Mt Etna *Protemnodon* populations, not the genera as a whole. This section should be written more succinctly.

- Section has been reworded to be more succinct and clearly state:

“Given the nature of sampling, isolated teeth examined here could not be easily identified to species level, so are grouped as local *Protemnodon* from Mt Etna. While Hocknull [40] identified three taxa within these sites and associated deposits at Mt Etna, these taxa do not conform to those recently described by Kerr et al. [36] so will require further comparative work before species-level identifications are possible. Therefore, we treat the individuals as members of *Protemnodon* with clear stratigraphic and geographic similarity meaning our results represent a site-specific analysis of *Protemnodon* individuals at Mt Etna, and not a study of the *Protemnodon* genus as a whole.”

Lines 502-506 and 536-539: These sections use two different listing styles (“1”, “2” and then “i”, “ii”, “iii”). I recommend choosing one style and keeping it consistent throughout the paper.

- Numbering system has been updated, now consistently using (1) … (2) etc.

**In addition, please address the following:**

1. Lines 36-38. I don't think any ecologist would argue that foraging range in eutherians is only dictated by body size, and your results and discussion invalidate this statement in any case (e.g. line 324). Please remove or reword accordingly.

- Sentence has been reworded “Our results suggest that, foraging ranges in marsupial megaherbivores may be more strongly associated with environmental quality rather than body mass.”
  - Statement suggesting that eutherian ranges are solely dictated by body range has been removed.

2. Lines 89-90. This sentence is incomplete.

- Reworded “Fossils **examined here were** recovered from the western side of Mt Etna Caves National Park (formerly a limestone mine) (Hocknull et al., 2007, Martinez, 2010).”

3. Lines 171-172. You draw attention to the size of one specimen, and clearly the size of the teeth are an important indicator of body size. What about the other teeth? You need to add dimensions of the teeth, or at the very least if this is no longer possible, at least indicate if they likely represent small, medium, or large *Protemnodons*.

- SAH measured teeth and provided notes on comparable taxon (Based on body mass & morphology). These have now been included in the **Supplementary Table B** and mentioned in text - Lines 171 onwards.
- “Despite the lack of species-level allocations, isolated teeth were grouped based on a comparison of their size to those measurements available in Kerr et al. (2024). Through this, individual teeth could be classified into two distinct size morphs: larger individuals, comparable in body mass to *Protemnodon viator* (Kerr et al., 2024) (n = 3), and smaller individuals comparable in body mass to *P. otibandus / P. tumbuna* (Kerr et al., 2024) (n = 5) (Table B in S1 Table).”
- Moreover, these differences of size are also incorporated in the discussion (when talking about the unique Sr composition of WIGL8550)
- “Measurements from WIGL8550 suggest the individual was comparable in size to *P. viator* (Kerr et al., 2024) marginally larger than other individual *Protemnodon* from Mt Etna Caves (S1 Table, Table B). While this may suggest this broader home range to adjacent geological substrates is driven by body mass, smaller foraging ranges, constrained to the Mount Alma Formation are observed in two additional individuals comparable to *P. viator* (Kerr et al., 2024). Therefore, a larger sample set with both small and large individuals of *Protemnodon* is required to determine whether broader scale dispersal is a product of increased body mass, or simply a unique behaviour observed in some highly vagile individuals. Based on the current dataset, we suggest that most *Protemnodon* from Mt Etna were non-vagile, though, broader scale to adjacent geological substrates was possible for some individuals.”

4. Line 191. You've not established that these represent eight individuals, only that they represent eight individual teeth. Please provide molar position for each molar, so you and the reader can determine if there is a possibility that different teeth from the same individual may be present in a unit in Table B, and provide MNIs per unit in the body of the text.

- Tooth numbers have been added to Table B which suggest that the MNIs examined here is 5. This number has now been updated in the main manuscript.

5. Line 204-205. This sentence is incomplete. But also, they don't represent a natural population, which has an ecological definition implying individuals within the population can reproduce. At best, you have individuals of a palaeocommunity.

- Mention of a ‘local population of *Protemnodon* at Mount Etna’ has now been removed.
- “Therefore, we treat the individuals as members of *Protemnodon* with clear stratigraphic and geographic similarity meaning our results represent a site-specific analysis of *Protemnodon* individuals at Mt Etna, and not a study of *Protemnodon* as a whole.”

6. Line 480 - not may, does account for some variation, based on the statistically significant results you reported!

- Sentence re-worded as follows “Phylogenetic linear regressions for Macropodidae suggest body mass accounts for some variation observed in home range trends (Fig 2), however, allometric relationships were significantly weaker than those observed in terrestrial herbivores from Tucker et al. (2014)”

7. Line 483 - first mention of *Petrogale* so spell out in full. More importantly, how were outliers established?

- Clear definition for outliers has now been included; individuals who fall outside the 99% confidence interval shown in Fig. 2 🡪 *Petrogale penicillate* spelt out in full.

8. Line 485 - as above.

- *Thylogale stigmatica* has been spelt out in full.

9. Lines 517-519. This statement is contradicted by your results (see Reviewer 2 comments). Also, to what does the Funck reference relate to?

- See response to Reviewer 2 comments above. Those section of text has been updated suggesting while there is variation between these specimens, this variation does not exceed that measured in the broader Mount Alma Formation (formation surrounding limestone). Moreover, isotope ratios in surrounding geological features are significantly higher/lower than those measured in most *Protemnodon* specimens which further supports the idea the individuals were localised.
- Funk Reference has been removed.

10. Lines 524-525. This sentence isn't grammatically correct. More importantly, it's not a fair comparison - one genus of marsupial vs all placentals. I'm sure placentals have their fair share of outliers, as indeed you point out further down.

- Sentence has been reworded to remove the comparison to all placentals
- “Limited home range in most *Protemnodon* individuals from the Mt Etna Caves deviate from that predicted based on body mass alone using our linear regression model based on body mass (11.6 ± 5.8 km2, Fig. 1).”

11. Lines 530-532 - but isn't it analgous to at least P. penicillata? And then in lines 547-550 you rely on them being analgous to form your hypothesis, despite saying they aren't. You need to be internally consistent with your arguments.

- The non-analogous statement was initially introduced as a comparison to mobile/nomadic, large-bodied extant species (*Osphranter rufus, Macropus giganteus* etc.), however, we acknowledge the wording contrasts hypotheses presented later in the paper.
- As such, this section has now been reworded, as follows:

1. We introduce the idea that observed ranges (From Sr isotope composition deviate from the modelled range)
2. We then suggest like extant Macropodidae, this may reflect the role of factors other than body mass, in dictating foraging range (particularly environment)
3. Following on with the environmental factor, we reference
4. We then reference conclusions from Kerr et al. 2024 suggesting that “species of *Protemnodon* exhibit a degree of ecomorphological variation suggestive of a broader array of ecological adaptations” – which supports the idea, external factors (not just body mass) may influence the extent of foraging ranges (This also introduces the lomomotory argumnent from Kerr discussed in lines 622 – 641)

- “This may suggest that like modern macropodids, the foraging ranges of *Protemnodon* from Mt Etna are not entirely dictated by body mass, and may be more strongly influenced by external factors including behaviour and environment (Fisher and Owens, 2000). These findings support previous interpretations of the *Protemnodon* genus from Kerr et al. (2024) suggesting ecomorphological variations across species were indicative of a broad array of ecological adaptations (including locomotory variations) across different palaeoenvironments. Therefore, the extinction risks for *Protemnodon* during the Pleistocene cannot be simplified using broad, genus-level interpretations. Instead, regionally specific baseline biologies need to be built using objective independent techniques.”

12. Line 593 - these animals aren't sedentary, which implies inactive, they just have a small range (although see point 9).

- Sentence has been rephrased, removed the term sedentary.

13. Line 618 - what do lemurs have to do with Australian megafauna? No clear link established

- Lemurs were mentioned, as they are a similar case study showing highly localised ranges in extinct megafauna.
- However, we acknowledge that these are arboreal frugivores in a distinctly different environment, and as such this mention has been removed.
- We now compare out results from Mt Etna to prior studies of Australian Megafauna from Wellington Caves and Bingara (Koutamanis et al., 2021) and the Darling Downs (Price et al., 2017b).

Figure 1 - show where in Australia this is. Merge with Figure 6. Add plant localities.

- Map showing Location of Mt Etna, relative to the rest of Australia has now been included in the bottom left-hand corner of the figure, this has also been merged with Fig 6 showing the 11.6 ± 5.8 km^2^ predicted foraging range for species of Protemnodon in red. Locations for vegetation sampling have been included and denoted by orange crosses.

Figure 2 - add species labels

- Species names have now been included in the figure.

**References**

ADAMS, S., GRÜN, R., MCGAHAN, D., ZHAO, J.-X., FENG, Y., NGUYEN, A., WILLMES, M., QUARESIMIN, M., LOBSEY, B., COLLARD, M. & WESTAWAY, M. C. 2019. A strontium isoscape of north-east Australia for human provenance and repatriation. *Geoarchaeology*.

FISHER, D. O. & OWENS, I. P. 2000. Female home range size and the evolution of social organization in macropod marsupials. *Journal of Animal Ecology,* 69**,** 1083-1098.

HOCKNULL, S. A., ZHAO, J.-X., FENG, Y.-X. & WEBB, G. E. 2007. Responses of Quaternary rainforest vertebrates to climate change in Australia. *Earth and Planetary Science Letters,* 264**,** 317-331.

KERR, I. A., CAMENS, A. B., VAN ZOELEN, J. D., WORTHY, T. H. & PRIDEAUX, G. J. 2024. Systematics and palaeobiology of kangaroos of the late Cenozoic genus Protemnodon (Marsupialia, Macropodidae). *Megataxa,* 11**,** 1-261.

KOUTAMANIS, D., MCCURRY, M., TACAIL, T. & DOSSETO, A. 2023. Reconstructing Pleistocene Australian herbivore megafauna diet using calcium and strontium isotopes. *Royal Society Open Science,* 10**,** 230991.

KOUTAMANIS, D., ROBERTS, G. L. & DOSSETO, A. 2021. Inter- and intra-individual variability of calcium and strontium isotopes in modern Tasmanian wombats. *Palaeogeography, Palaeoclimatology, Palaeoecology,* 574.

MARTINEZ, S. 2010. *Palaeoecology of the Mount Etna bat fauna, coastal Eastern Queensland.* Queensland University of Technology.

PRICE, G. J., CRAMB, J., LOUYS, J. & FENG, Y.-X. Palaeontology of northeastern Australian caves. Proceedings of the 17th International Congress of Speleology, 2017a. Australian Speleological Federation Incorporated Sydney, 22-25.

PRICE, G. J., FERGUSON, K. J., WEBB, G. E., FENG, Y.-X., HIGGINS, P., NGUYEN, A. D., ZHAO, J.-X., JOANNES-BOYAU, R. & LOUYS, J. 2017b. Seasonal migration of marsupial megafauna in Pleistocene Sahul (Australia–new Guinea). *Proceedings of the Royal Society B: Biological Sciences,* 284**,** 20170785.

RADLOFF, F., MUCINA, L., BOND, W. & LE ROUX, P. 2010. Strontium isotope analyses of large herbivore habitat use in the Cape Fynbos region of South Africa. *Oecologia,* 164**,** 567-578.

TUCKER, M. A., ORD, T. J. & ROGERS, T. L. 2014. Evolutionary predictors of mammalian home range size: body mass, diet and the environment. *Global Ecology and Biogeography,* 23**,** 1105-1114.

ZIELIŃSKI, M., DOPIERALSKA, J., KRÓLIKOWSKA-CIĄGŁO, S., WALCZAK, A. & BELKA, Z. 2021. Mapping of spatial variations in Sr isotope signatures (87Sr/86Sr) in Poland—Implications of anthropogenic Sr contamination for archaeological provenance and migration research. *Science of the Total Environment,* 775**,** 145792.
