# Supplementary material for: Megafauna mobility: Assessing the foraging range of an extinct macropodid from central eastern Queensland, Australia
Source: PLoS One. 2025 Apr 23;20(4):e0319712. doi: 10.1371/journal.pone.0319712 (PMC12017834; doi:10.1371/journal.pone.0319712)
Supplement: Attachment — Submitted filename: Response to Reviewers.docx [file pone.0319712.s015.docx]

Reviewer #1:

Introduction
Lines 52-54: Mentions a “significant amount of research” but cites none of it. Be sure to add in relevant citations here for studies that both focus on mechanisms, and those that focus on life history. There are several on both accounts, so be sure to cite them.

References for these points have now been included.

Lines 56-57: “a large body size provides opportunities for greater geographical range…” greater than what? Need to be clear.

Sentence has been made clearer, “As observed with extant megafaunal herbivores, increases in body mass are positively correlated with the extent of an individual’s home range”.

Lines 57-60: “Whilst body mass is considered a strong indicator in placental mammals [5], external factors including habitat type [6] – specifically relationships between opened and closed habitats – may also play a role in dictating the extent of home range.” I’m having trouble understanding the phrasing here. Suggest a re-write to be clear to emphasise exactly what “relationships” you’re talking about.

Variable home ranges in open and closed habitats are unpacked in the following sentences, so the mention of these have removed for clarity.

Lines 60-64: Again, I’m having trouble understanding the arguments here. The paragraph opens with the specific research question about the extinction of Quaternary megafauna, but later deviates to ecological aspects of South American and African extant communities. It’s unclear what the link to late Quaternary extinctions in Australia is here.

Introductory sentences about quaternary megafauna and research question have been separated into its own paragraph. Then, the following paragraph discusses foraging range as a key aspect of life history. In the original version, ecological aspects of extant South American and African herbivores were included, referencing specific case studies where comparably smaller home ranges were reported in closed-forest species when compared to open-forest species of a similar body mass. We acknowledge that these mentions may cause some confusion, so the specific cases have been removed.

“Specifically, species residing in closed-forest habitats often have smaller home ranges than species of a comparable body mass residing in open-habitats”.

Lines 71-76. I’m sorry, but I’m having trouble making of the phrasing through here and the inference with respect to geology, botany, or feeding activities of herbivores.

Section has now been phrased in a better manner. The initial sentence unpacks the factors driving unique isotopic ratios in geological substrates. The following sentence then describes how unique Sr composition from geological substrates are transferred into plants and then organisms.

“Unique isotopic signatures are transferred to plants through the weathering of substrates, and into trophic chains through the consumption of plant material [18, 19]. A large atomic mass and small relative difference in masses between isotopes means they are less susceptible to kinetic fractionation associated with biological processes [20]. Consequently, the strontium isotope composition of an individual reflects dietary intake and the isotopic composition of its environment [21]”

Lines 78-79: “Strontium isotope ratios in terrestrial consumers are predominately derived from diet.” No, ratios are something that are measured and not derived from diet. I know what you mean, but you need to think about phrasing. Keep in mind too that the accumulation of strontium isotopes in biological tissues is not from ‘diet’, but ‘feeding activities’ (‘diet’ is something we observe but is not the activity itself).

See previous comment, in reworking this section, the specific sentence has been removed and instead replaced with the following “Consequently, the strontium isotope composition of an individual reflects dietary intake and the isotopic composition of its environment [21]”

Lines 88-90: Need to clearly state what the “trends” in mineralisation are- i.e., as related to ontogeny.

This was initially mentioned in the following paragraph. For the sake of clarity, this section has been re-ordered and explicitly stated.

“In macropodids enamel mineralisation in incisors (I) and early molars (first and second molars, M1 & M2) coincides with weaning (Brookman and Ambrose, 2012) whilst later forming molars (third and fourth molars, M3 & M4) post-dating weaning (Brookman and Ambrose, 2012, Brookman and Ambrose, 2013). Therefore, bio-available Sr from early forming teeth will reflect dietary intake of an individual’s mother, while later-forming molars reflect initial dietary independence.”

Lines 100-101: The position of the genus is not poorly understood as implied in this sentence. It’s a sister taxon to Macropus and member of the same family; this is an inference that has been unchallenged for the past 50+ years if not more and is one of the principal findings of the study that is cited here (reference 35- Cascini et al.).

Inference that the position of the genus is poorly understood has now been removed.

Lines 91-106: This paragraph needs attention. It covers all sorts of themes including introduction of a study site, geography, climate change, extinction, a specific taxon, its taxonomic position, then merging into palaeobiology touching on dentition and post-cranial functional morphology. It’s very hard to follow. Best to think about paragraph structure and their themes.

Section has now been broken down into smaller paragraphs covering the individual themes. The first paragraph covers the introduction to the study site and the current understanding of chronology and ecosystem shifts experienced at Mt Etna Caves. A new concluding sentence has been added, highlighting that the study is focused on rainforest deposits.

Line 107: “Given megafaunal macropodids dominate Australia’s fauna…” what does this mean? Macropodids are the largest-bodied endemic herbivores today, but that is not true across geologic time. The largest-bodied mega-herbivores were the diprotodons and ancestors for most of the Quaternary, and all of the 25-odd million years before that.

Poor choice of words, sentence was meant to reflect the idea that macropodids are a major part of Australia’s faunal diversity, both today and in the Late Pleistocene. The phrasing of this sentence has now been updated.

“Given macropodids form a major component of Australia’s fauna, understanding the past foraging ranges…”

Materials and Methods

Lines 127-147. You need to cite the primary literature for this source of geological information rather than an unpublished thesis. Most of the synopsis presented in this section does not appear to have come from Deer’s 2011 thesis.

Geological information was derived from published datasets provided by the QLD government, Department of Resources. This dataset has now been cited in this section.

Lines 155-156. The first supplementary table referred to in the manuscript is “Supplementary Table S16”. You need to have data cited in order through the text from 1 to N, not randomly starting at 16. In any case, there is no Table 16 in the Supplementary files- it only goes to S14 so I’m having trouble understanding what you’ve done.

This issue has now been remedied (updated to Table A in Table S1). Also has been remedied with all forthcoming tables.

Lines 156-157: “Data were obtained from Tucker et al. [5] and Goldingay [48].” What type of data? Need to be specific if you are using data from multiple sources. Does this pertain to mass and home range?

Sentence has now been moved to the beginning of this paragraph for clarity. Reflects that Home range (km2) and body mass (kg) data was acquired from these two publications

“Using data presented in Tucker et al. (2014) and Goldingay (2023), estimates of body mass (in kg) and mean home range (in km^2^) were collated for 17 extant species belonging to the Macropodidae family”

Looking at Supplementary Table 14, there are numerous and out-dated taxonomic errors in there. If this is the primary source of the manuscript and meant to be “S16”, then it needs to be substantially revised.

Taxonomic errors have now been revised for this table.

Lines 157-159. Need to state what the protocols were, and more specifically, what this means for the submitted study. What is the “weighted average” calculated from?

Following Tucker et al. for the regression model, we require a single value for body mass (kg) and predicted home range (Km^2^). So, in instances where the literature contained multiple estimates of home range and/or body mass, we generated a singular mean value incorporating all published results in proportion to their associated error.

Lines 159-160. “Phylogeny included in the model...” What model? I’m having trouble understanding the rationale here. A database of extant macropodids was collated (Line 155-157) but the rest of the paragraph does not explain why or how those data were used except for something about an R analysis. You need to be explicit in how you can use this data to make foraging range estimates in a meaningful way.

Phylogenetic method and R analysis have now been moved to a separate paragraph. Final Sentence of this initial paragraph now unpacks how body mass and home range data will be used, and the rationale behind this use.

Lines 165-169. The paper here cites reference 34 (Kerr et al.) as a source of information, but the taxonomy presented in the manuscript does not follow Kerr et al. at all, at least for some of the species mentioned. The authors need to be clear in their methodology.

Mentions of species that do not follow taxonomy presented in Kerr et al. have now been removed from the paper. As mentioned in the introduction, *Protemnodon* specimens from Mt Etna Caves, do not conform to the new taxonomy defined in Kerr et al., so we are unable to group specimens here into a specific species. As WIGL8550 (an M4 molar) has similar dimensions to M4 molars in *P. viator* and *P. mamkurra* described in Kerr et al. we can assume that Protemnodon from Mt Etna Caves exhibit a similar body mass – assuming tooth size is comparable to body size – and therefore fall within the 50 – 170 kg range included in the

Furthering this point, an additional paragraph has now been added to this section acknowledging that from our current understanding, species of Protemnodon are vastly different to extant macropodids & these modelled home ranges may not be applicable. However, they still reflect predicted range extent for a 50 – 170 kg Macropodiforme and therefore, provide a means of testing whether Protemnodon conform to body-mass home range relationships observed in extant macropodids, or whether they are doing something incredibly different.

Lines 173-176. Mentions that some samples were chosen for analysis but doesn’t say what the analysis is. Need to also specifically say what the teeth numbers were, i.e., m1, m2, etc.

Mention of in-situ laser ablation has now been added to highlight the type of analysis undertaken.

-----

At this stage, I can’t go much further with this review. I’ve been bogged down in the Introduction and Methods for some time now, and there are numerous things that already need to be addressed for this study to be publishable. I really encourage the authors to spend more time revising the manuscript. I think that there is something really good here and is quite novel, but the significance of the study is lost in the framework as presented. I’m not going to recommend publication at this stage but urge the authors to not give up on what could be an excellent scientific contribution.

For what it’s worth, I’ve briefly read over the rest of the manuscript. Some basic things to note include:
1) Cite and integrate supplementary data properly, and present it logically and in order following the main text.

All supplementary material has been updated, following the same chronological order presented in the main text.

2) **I appreciate that Sr data of local geological formations may not be available, but I strongly do not recommend using Sr values of apparently similar rock types from other continents as substitutes in the construction of an isoscape- this is methodologically unsound for a variety of reasons** and ultimately may affect the overall findings (Lines 293-295 and Supplementary Table 5- and noting too, most of the references in this supp. table are to a bibliographic database of the literature, not the actual studies themselves that published the Sr data);

We note that in previous studies of strontium isotopes and foraging range in the Australian setting have been published without Sr data from local geological formations (Price et al., 2017, Koutamanis et al., 2023). However, given that this methodology is unsound, we have undertaken additional analyses of ^87^Sr/^86^Sr isotope ratios in overlying vegetation. Vegetation analyses have been completed for 24 vegetation samples covering 11 distinct geological units highlighted in figure 1. As these distinct geological units beyond the Mt Alma Formation show significantly higher/lower Sr composition, the interpretation of restrictive foraging ranges still stands.

Descriptions of methodology for bio-available Sr sampling have now been included in the supplementary material.

3) You need to better consider the implications of the new ages and not overreach. This has major implications for the study’s findings (e.g., Lines 415-427) that play into aspects of understanding the timing of climate change / habitat loss / extinctions in the region. The new interpretation that the mid-Bruhnes climate change event caused rapid impacts on Mt Etna ecosystems is not supported by the data presented.

We appreciate the comments on our age data and have now updated the interpretations for individual deposits.

a. For example, the oldest previous minimum age for site QML1311H is 454+-48 ka (U-series dated calcite infill of bone), but the burial age from the new luminescence dating is around 100 millennia younger. There must be something wrong somewhere here- do not ignore this discrepancy.

For QML1311H, we now acknowledge this pre-existing oldest minimum age (454 ± 48 ka), however, we suggest that since all other U-series ages reported in Hocknull et al. conform with our U-series ages presented here, it is likely this one sample is an outlier, potentially reflecting a reworked calcite originating in an older deposit. Excluding this date, we then suggest fossil deposition in QML1311H occurred between 285 – 304 ka (Based on the oldest available minimum age from u-series dating and the TT-OSL dates presented here).

b. For QML1311C/D, the previous maximum age (326+-22 ka from a U-series dated basal flowstone) is consistent with the new burial luminescence age (291+-28.3 ka). The new U-series ages of teeth are much younger (as young as 222+4/-3 ka) but are minimum ages; they are not inconsistent with the burial age, but you then can’t make the argument that the deposit dates to as young as 210 ka (Lines 404-405). There is no evidence for that at all; this is a misinterpretation of the data and premise of the dating approach being that they are not a closed system.

For QML1311C/D we have removed the argument suggesting fossil deposits may be as young as 210 ka. Instead we take the oldest minimum age from u-series dating (280 +21/-18ka) and the maximum age defined in Hocknull et al. to suggest fossil deposition is likely to have occurred between 280 – 330 ka. (This better refines previous estimates for this deposit which simply suggested <330ka)

c. For QML1384LU, the youngest minimum age comes from the previously U-series dated calcite infill in bone (332 +-14 ka; although there is a typo in the manuscript where it reports this age as “323”- Line 412). The new U-series dates on teeth are minimum ages (as young as 213+-13/9 ka) and are not inconsistent with the minimum age of the calcite infill, but you can’t then argue that the deposit is as young as 210 ka (Line 416)- this cannot possibly be true. The minimum age of the fossil cannot be younger than the oldest minimum age of the deposit- in this case 332 +-14 ka dated from calcite infill in bone. To argue this is another misinterpretation of the data and approach.

For QML1384LU, we acknowledge our U-series ages are minimum ages, and given this, the oldest minimum age from Hocknull et al. still remains

4) The underlying assumption is that species within the study taxon, Protemnodon, are directly analogous, biologically and ecologically speaking, with extant macropodines (e.g., Methods, Results, Discussion). However, that interpretation deviates significantly with current palaeobiological understandings of species within the genus that show that they are unlike anything extant today (Janis et al. 2020 J Mamm. Evol.; Jones et al. 2022 J. Mamm. Evol.; Kerr et al. 2024 Megataxa; papers of which are cited in the manuscript but that general finding is ignored). I strongly encourage the authors to better consider the palaeo-biological/ecological significance of the study taxon in order to best interpret it.

Methods, results and discussion have now been modified to reflect this notion that *Protemnodon* are vastly different than extant macropodids today. However, as we know so little about life history of *Protemnodon*, we need to use biological and ecological traits in extant macropodines as a point of comparison. Using this comparison, we prove that foraging behaviour significantly deviates from what is expected of a large-bodied macropodid which in turn supports the current palaeobiological interpretation that species of *Protemnodon* are unlike anything extant today.

For the methodology (specifically modelling home ranges) we now acknowledge that *Protemnodon* are unlike extant macropodids but suggest our linear regression model provides a rough estimate of home range in a 50 – 170 kg macropodid. If *Protemnodon* from Mt Etna Caves do not conform to these estimated home ranges, we can assume that this dissimilarity supports current palaeobiological understanding that species of *Protemnodon* are vasty different to extant macropodids.

Reviewer #2: This study Is the result of original research and as far as I can discern results have not been reported elsewhere. Methods are well described and rigorously performed to a high standard. The article is intelligible and written in standard English. Data availability guidelines are also met. However, conclusions are not fully supported by the data as follows.

I have two concerns. First, the authors indicate that Sr values associated with Mt Etna Protemnodon suggest short scale movements (Lines 448-449) but “…to reliably estimate the extent of these, a more extensive surveying of bio-available strontium and an isoscape quantifying spatial variability of 87Sr/86Sr will be required” (Lines 452-454). A background Sr dataset is always required for such a study to meet standards of research integrity. Whereas Mt Etna limestone 87Sr/86Sr data were available, using “**common…values for corresponding rock types” (Line 294-295) does not meet this standard**. A Sr sampling regime that encompasses the estimated home range of Protemnodon based on the taxon’s body mass is needed to support the argument that Protemnodon’s foraging range was smaller than expected, thus not scaled to its body mass but “…likely due to a unique combination of behaviour, diet and/or locomotion” (Lines 40-41, 463-466).

See comments above in relation to (2) raised by Reviewer 1. Briefly, we acknowledge that using common values for local lithology is a laxed approach. However, this was following previously published Australian case studies, Price et al. (2017) and Koutamanis et al. (2023). To amend this, we have undertaken additional sampling of bioavailable Sr in 24 vegetation samples covering 11 geological units defined in Figure 1 of the manuscript. ^87^Sr/^86^Sr isotope ratios in adjacent geological features are significantly higher/lower than those measured in *Protemnodon* enamel meaning arguments for a relatively localised macropodid still stand.

Secondly, the authors argue that Protemnodon was foraging in a “biodiverse rainforest ecosystem” characteristic of Mt Etna prior to 210 ka. Thus, Protemnodon was likely browsing not grazing and a smaller than expected foraging range was not surprising given that “…Mt Etna’s tropical rainforest environment provided a productive ecosystem where dietary needs could be met with relatively small foraging ranges” (Lines 476-477). Yet, no data are provided in support of Protemnodon’s hypothesized dietary intake. Such evidence would be useful adding credence to the author’s explanation for a smaller than expected foraging range relative to body mass. **Tooth enamel sampled for Sr yielded very little evidence of diagenetic alteration, (Supplemental Materials.S3). Thus, Protemnodon enamel can also be sampled for hydroxyapatite stable carbon isotope values. Methods are well established and d13C enamel data are commonly considered reliable even in mya settings.** If Protemnodon was foraging in a closed canopy rainforest enamel d13C values will be diagnostic of that setting, more negative than expected of a C3 open foraging environment given the recycling of atmospheric CO2. Results will either support or call into questions the authors’ argument that Protemnodon’s foraging range was determined by climate rather than scaled to body mass. A wider sampling of the Sr isoscape is needed to confirm that argument.

We acknowledge that carbon isotope analyses would significantly strengthen arguments for a browsing, rainforest dwelling *Protemnodon.* But with the additional analyses of ^87^Sr/^86^Sr in local vegetation, carbon isotopic analyses were not feasible at the present moment. Given this, at present, arguments surrounding diet remain reliant upon; inferred browsing in closed forest species defined in Kerr et al., and morphology of *Protemnodon* from Mt Etna Caves with low-crowned molars suggesting individuals from Mt Etna exhibited the greatest browsing potential across the broader genus. In the discussion, we acknowledge that the current argument is a hypothesis based on current understanding of morphology, and additional proxies are necessary to confirm or challenge this idea of browsing. To conclude, we agree that dietary proxies (carbon isotopes) would be an excellent addition, and provide strong empirical support for the proposed browsing diet. Whilst including these is beyond the scope of this study, we appreciate the suggestion and will undertake analyses of C isotopes in future studies expanding on this preliminary work.

**Additional Journal Requirements:**

When submitting your revision, we need you to address these additional requirements.

1. Please ensure that your manuscript meets PLOS ONE's style requirements, including those for file naming.

File Names have now been remedied.

2. In your manuscript, please provide additional information regarding the specimens used in your study. Ensure that you have reported human remain specimen numbers and complete repository information, including museum name and geographic location.

Statement regarding permits has now been included in the methods section (For fossil specimens) and in the supplementary material for vegetation sampling. Additional information regarding the specimens. Associated WIGL numbers will be logged with the Queensland Museum.

3. In your Methods section, please provide additional information regarding the permits you obtained for the work. Please ensure you have included the full name of the authority that approved the field site access and, if no permits were required, a brief statement explaining why.

See previous comment – permits have now been included for relevant methodology.

4. Please note that funding information should not appear in the Acknowledgments section or other areas of your manuscript. We will only publish funding information present in the Funding Statement section of the online submission form. Please remove any funding-related text from the manuscript.

Reference to funding has now been removed from the acknowledgements.

5. When completing the data availability statement of the submission form, you indicated that you will make your data available on acceptance. We strongly recommend all authors decide on a data sharing plan before acceptance, as the process can be lengthy and hold up publication timelines.

All data has been made available in the attached ‘S1 Table.xls’ file included in the submission.

6. Please amend either the title on the online submission form [(macropodid, from) via Edit Submission] or the title in the manuscript so that they are identical.

Title has now been amended in the submission portal. Both should read “Megafauna mobility: assessing the foraging range of an extinct macropodid from central eastern Queensland, Australia.”

7. We note that Figures 1 and 6 in your submission contain map images which may be copyrighted. All PLOS content is published under the Creative Commons Attribution License (CC BY 4.0), which means that the manuscript, images, and Supporting Information files will be freely available online, and any third party is permitted to access, download, copy, distribute, and use these materials in any way, even commercially, with proper attribution. For these reasons, we cannot publish previously copyrighted maps or satellite images created using proprietary data, such as Google software (Google Maps, Street View, and Earth). For more information, see our copyright guidelines: <http://journals.plos.org/plosone/s/licenses-and-copyright>.

We require you to either (1) present written permission from the copyright holder to publish these figures specifically under the CC BY 4.0 license, or (2) remove the figures from your submission:

We have followed up with the support and have received the following: As GeoResGlobe datasets are under the Creative Commons Attribution 4.0 licence, they may be adapted and redistributed for any purpose. Attributions have now been updated to match the template provided from the original figure owner.

Additionally, for S2 Figure, permission to reproduce has been obtained from the initial publisher (EPSL, Elsevier) (See attached upload – **Elseiver_Copyright**) and appropriately referenced in the figure caption.

8. Please include captions for your Supporting Information files at the end of your manuscript, and update any in-text citations to match accordingly. Please see our Supporting Information guidelines for more information: <http://journals.plos.org/plosone/s/supporting-information>.

Captions for supporting information is now included at the end of the manuscript. As highlighted in an earlier reviewer comment, these have all been re-ordered to match the main text.

**References**

BROOKMAN, T. H. & AMBROSE, S. H. 2012. Seasonal variation in kangaroo tooth enamel oxygen and carbon isotopes in southern Australia. *Quaternary Research,* 78**,** 256-265.

BROOKMAN, T. H. & AMBROSE, S. H. 2013. Kangaroo tooth enamel oxygen and carbon isotope variation on a latitudinal transect in southern Australia: implications for palaeoenvironmental reconstruction. *Oecologia,* 171**,** 403-416.

GOLDINGAY, R. L. 2023. Movement patterns, home range and habitat selection of Australasian marsupials. *American and Australasian Marsupials: An Evolutionary, Biogeographical, and Ecological Approach.* Springer.

KOUTAMANIS, D., MCCURRY, M., TACAIL, T. & DOSSETO, A. 2023. Reconstructing Pleistocene Australian herbivore megafauna diet using calcium and strontium isotopes. *Royal Society Open Science,* 10**,** 230991.

PRICE, G. J., FERGUSON, K. J., WEBB, G. E., FENG, Y.-X., HIGGINS, P., NGUYEN, A. D., ZHAO, J.-X., JOANNES-BOYAU, R. & LOUYS, J. 2017. Seasonal migration of marsupial megafauna in Pleistocene Sahul (Australia–new Guinea). *Proceedings of the Royal Society B: Biological Sciences,* 284**,** 20170785.

TUCKER, M. A., ORD, T. J. & ROGERS, T. L. 2014. Evolutionary predictors of mammalian home range size: body mass, diet and the environment. *Global Ecology and Biogeography,* 23**,** 1105-1114.
